# Improved 93-11 Genome and Time-Course Transcriptome Expand Resources for Rice Genomics

**DOI:** 10.3389/fpls.2021.769700

**Published:** 2022-01-21

**Authors:** Sen Wang, Shenghan Gao, Jingyi Nie, Xinyu Tan, Junhua Xie, Xiaochun Bi, Yan Sun, Sainan Luo, Qianhui Zhu, Jianing Geng, Wanfei Liu, Qiang Lin, Peng Cui, Songnian Hu, Shuangyang Wu

**Affiliations:** ^1^Shenzhen Branch, Guangdong Laboratory for Lingnan Modern Agriculture, Genome Analysis Laboratory of the Ministry of Agriculture, Agricultural Genomics Institute at Shenzhen, Chinese Academy of Agricultural Sciences, Shenzhen, China; ^2^State Key Laboratory of Microbial Resources, Institute of Microbiology, Chinese Academy of Sciences, Beijing, China; ^3^Gregor Mendel Institute, Austrian Academy of Sciences, Vienna, Austria

**Keywords:** time course transcriptome, alternative splicing, *waxy*, 93-11, chromosome level

## Abstract

In 2002, the first crop genome was published using the rice cultivar 93-11, which is the progenitor of the first super-hybrid rice. The genome sequence has served as a reference genome for the *indica* cultivars, but the assembly has not been updated. In this study, we update the 93-11 genome assembly to a gap-less sequence using ultra-depth single molecule real-time (SMRT) reads, Hi-C sequencing, reference-guided, and gap-closing approach. The differences in the genome collinearity and gene content between the 93-11 and the Nipponbare reference genomes confirmed to map the *indica* cultivar sequencing data to the 93-11 genome, instead of the reference. Furthermore, time-course transcriptome data showed that the expression pattern was consistently correlated with the stages of seed development. Alternative splicing of starch synthesis-related genes and genomic variations of *waxy* make it a novel resource for targeted breeding. Collectively, the updated high quality 93-11 genome assembly can improve the understanding of the genome structures and functions of *Oryza* groups in molecular breeding programs.

## Introduction

For decades, the rice cultivar 93-11, which is one of the main *indica* cultivars grown across China, has served as a reference genome for molecular breeding research (Yu et al., 2002). Recently, with the development of sequencing technology and the dramatic decrease in its cost, several chromosome-level assemblies of various rice cultivars have been reported, including telomere to telomere assemblies of ZS97 and MH63 (Du et al., 2017; Choi et al., 2020; Song et al., 2021). The assembly data reveals the complex genetic diversity that give rise to desirable domestication traits. As 93-11 is the parental cultivar of super-hybrid rice LYP9, which is widely grown across China, a high-quality reference genome of 93-11 is necessary for future molecular breeding of super-hybrid rice. Meanwhile, the 93-11 genome is the historical reference genome for *indica*; therefore, an upgrade may also facilitate evolutionary studies of these cultivars.

The amylose content (AC) is the most important factor that affects the taste and cooking quality of rice. The AC phenotype is mainly determined by the starch synthesis pathway. GBSSI (granule-bound starch synthase I), which is encoded by the *waxy* (*Wx*), is essential for the biosynthesis of amylose in rice (Vrinten and Nakamura, 2000; Tian et al., 2009). The evolution of the *Wx* largely contributed to improving the eating and cooking quality of rice, and its allelic variants (*Wx*^a^, *Wx*^b^, *Wx*^in^, *Wx*^op^, and *Wx*^mp^) affected the AC of rice thereby affected consumer preferences (Zhang et al., 2019). The splicing form of the *Wx* and its related gene regulatory network contribute to the phenotypic selection in rice. Two Ser/Arg-rich proteins, Os-RSp29 and Os-RSZ23, can enhance, splice, and alter the acceptor splice site in the first intron of *Wx*^b^ (Isshiki et al., 2006). The Prp1 protein Du1 (dull endosperm1) affects the splicing efficiency of *Wx*^b^ and regulates starch biosynthesis (Zeng et al., 2007). In addition, the transcription factors OsBP-5, OsEBP and OsbZIP58 are involved in the regulation of *Wx* expression and starch biosynthesis (Wang et al., 2013). The metabolism involved in the amylose synthesis pathway is complicated and is a typical quantitative genetic trait. Recent studies have documented that the large structural variations between *indica* and *japonica* groups, which contribute to the cost of domestication of rice (Kou et al., 2020). Thus, it is necessary to genotype the agricultural traits of individual genomes.

In this study, we present the high-quality chromosome level genome sequence of the 93-11 cultivar using Illumina short pair-end reads, PacBio long reads, and Hi-C contact reads. This high-quality genome enabled us to investigate the structural variations between the *indica* and *japonica* groups. We also designed a continuous transcriptomic sampling experiment from 4 to 16 days after pollination (DAP) to investigate the gene expression pattern during seed development, as well as the gene regulation network of starch synthesis, which is conducive to the development of follow-up breeding work. This assembly and the related sequencing data provide a valuable complement to the rice study community and contribute to the subsequent breeding research.

## Materials and Methods

### Plant Material

All cultivars, including 93-11, were collected from paddy fields in Beijing, China (Supplementary Table 1). Young leaves and seed samples collected at DAP 9 from Nipponbare and 12 other cultivars, as described in the Supplementary Table 1, were used to construct paired-end DNA-seq and RNA-seq libraries, respectively. For time course analysis, we collected samples during seed development until the full grain stage (from 4 to 16 DAP). All samples were washed using sterile physiological saline (37°C), snap-frozen in liquid nitrogen, and stored at −80°C.

### Sample Preparation

Genomic DNA was extracted from young leaves using TRIzol to construct the paired-end library, single molecule real-time (SMRT) library, and Hi-C library, by following the manufacturer’s protocol. The quality and quantity of the DNA were evaluated using a 0.8% agarose-gel electrophoresis, a NanoDrop micro-spectrophotometer (NanoDrop Technologies, Wilmington, DE, United States), and a Qubit ^®^ 3.0 Fluorometer (Thermo Fisher Scientific, Carlsbad, CA, United States), respectively.

Total RNA was extracted using the Huayueyang Plant RNA Extraction Kit (GK0416; Huayueyang, Beijing, China) according to the manufacturer’s instructions. The RNA quality and quantity were examined using an electrophoresis agarose gel and a NanoDrop micro-spectrophotometer (NanoDrop Technologies), respectively.

### Genome Sequencing

Sequel ^®^ Sequencing Kit 2.1 (PacBio) was used to construct an SMRT library, and high-quality long reads were generated using an RSII system. For Hi-C, libraries were performed according to a previously described method (Wang et al., 2020; Wu et al., 2020), via *Hin*dIII digestion and sequencing using a Hiseq X platform (Illumina) with an average depth of 69 ×. Poly (A) strand-specific libraries were constructed using the KAPA Stranded mRNA-Seq Library Preparation kit (KK8421; Roche, Pleasanton, CA, United States). All libraries were sequenced using an Illumina Hiseq 4000 sequencing system.

### Genome Assembly

Subreads with an ultra-depth of 196 × that combined the dataset from a previous study (Zhang et al., 2018) were used to perform the *de novo* assembly using CANU (version 1.8) (Koren et al., 2017) with the parameters “minReadLength = 3,000, correctedErrorRate = 0.030, batOptions = -dg 3 -db 3 -dr 1 -ca 500 -cp 50.” The raw contigs were further corrected using the Illumina pair-end reads using Pilon (version 1.23) (Walker et al., 2014), and the haplotypic duplication contigs were removed using Purge Haplotigs (version 1.1.1) (Roach et al., 2018).

To construct a high-quality chromosome level assembly, we further carried out the following steps (Supplementary Figure 1): (I) All the contigs were anchored on the chromosome using the Hi-C reads by Juicer (parameters: -s *Hin*dIII) and 3d-dna pipelines (parameters: -r 0 -i 50000 -m haploid) (Durand et al., 2016; Dudchenko et al., 2017). (II) Misjoins of the interaction map were corrected in JBAT (Juicebox Assembly Tools). (III) The accurately identification of telomere and centromere sequences. The tandem repeats (TRs) in the contigs were identified using the TRF program (Benson, 1999). The ends of the contigs containing the tandem repeat motif AAACCCT or AGGGTTT were considered as telomere regions, and these contigs were located at the ends of the pseudo-chromosomes. Hmmsearch (version 3.3) (Eddy, 1998) with the default settings was used to detect the location of the centromere sequences in the genome using an hmm file built from a file containing 155–165 CentO satellite sequences. The contigs containing TRs (unit: the satellite sequence) with a length of more than 10 kb were considered as centromere contigs and located in the middle of the pseudo-chromosomes. (IV) Self-alignment of all the contigs was performed by Mummer (version 4.0) with the default settings to determine the contig overlap and merge two adjacent contigs. (V) Long reads were aligned to all the contigs using Minimap2 (version 2.17-r941) to detect the adjacent contigs that shared the same long reads. If two adjacent contigs overlapped with the two ends of long reads, respectively, then the gap between the two contigs were closed using the reads. (VI) Mummer (version 4.0) (Delcher et al., 2003) with the default setting was used to align the contigs on the three genomes [Nipponbare (Kawahara et al., 2013), ZS97, and MH63 (Song et al., 2021)] to detect the potentially adjacent contigs. If one unlocated contig is adjacent to a contig in the interaction map along as supported by PacBio reads, and the unlocated contig was put back into the map. Steps IV and V were performed again to close gaps. (VII) Long reads were aligned to the polished contigs to detect sequence continuity. Finally, we manually checked the errors and finalized the chromosome.

The completeness of the assembly was assessed using the Benchmarking Universal Single-copy Orthologs (BUSCO) pipeline (version 5.0.0) (Seppey et al., 2019) with the embryophyta_odb10 dataset, which contains 1,614 highly conserved genes. Merqury was used for the assembly consensus accuracy estimation (Rhie et al., 2020).

### Repeat Annotation

The specie-specific repetitive sequence library and transposable element (TE) library were identified using RepeatModeler (version 1.0.5) (Flynn et al., 2020) and Extensive *de novo* TE Annotator (EDTA) (Su et al., 2021), respectively. Mummer (version 4.0) the default parameters was used to align the sequences from the specie-specific repeat library (REL) to transposable elements of the TE library (TEL). The sequences in the REL with a coverage ratio of 0.5 to a sequence in the TEL were filtered, and the TEL and REL with remained sequences were merged to construct a non-redundant repetitive sequence library. The dispersed repeats and TRs in the 93-11 genome were annotated by RepeatMasker (version 4.0.5) (Tarailo-Graovac and Chen, 2009) with the curated library.

### Gene Annotation

Protein-coding gene annotation was performed using a strategy integrating *de novo* prediction, transcriptome-based prediction, and homology-based prediction. Hisat2 (version 2.1.0) and StringTie (v2.1.2) were used to align the RNA-seq reads and assemble the transcripts (Pertea et al., 2016). The complete open reading frames (ORFs) of the transcripts from the 13 stages were identified to generate high-confidence gene models using the TransDecoder pipeline (version 5.5.0) (Haas et al., 2013). Augustus (version 3.2.2) (Keller et al., 2011) was used to predict gene models based on the repeat-masked and unmasked genomic sequences with the parameters ‘‘-s rice,’’ respectively. The protein sequences obtained from Nipponbare were aligned to the 93-11 genome using GenomeThreader (version 1.7.)^1^ with the parameters “-species rice.” The gene models obtained from the three prediction methods were integrated to generate a consensus gene set using GFFRead (version 0.11.6) (Pertea and Pertea, 2020) and an in-house Perl script. The gene structure was visualized via manual polishing using Integrative Genomics Viewer (IGV) (Robinson et al., 2011), and 3,215 genes were corrected based on the structural information, completeness of genes, and similarities to proteins of other species.

Gene functions were further annotated using protein-protein basic local alignment search tool (BLASTP) (e-value: 1 × 10^–5^) against the non-redundant protein database (NR) (Pruitt et al., 2005) and Kyoto Encyclopedia of Genes and Genomes (KEGG) database^2^. Interproscan (v5.21) (Quevillon et al., 2005) was used to identify the protein domains and annotate the gene ontology (GO) protein function.

### Structural Variation Identification

Mummer (version 4.0) (Delcher et al., 2003) with the default parameters was used to align the 93-11 genome to the Nipponbare genome. The alignment was filtered using delta-filter implemented in Mummer with the parameters “-i 90 -l 10000,” and the filtered alignment was plotted and displayed using mummerplot. The alignment was also filtered using delta-filter with the parameters “-i 80 -l 1000” to analyze the syntenic regions and genome structural variation between the 93-11 and Nipponbare genomes.

### Homolog Comparison

All-versus-all BLASTP (e-value: 1 × 10^–5^) was used to calculate the pairwise similarities of the protein sequences between the 93-11 and Nipponbare genomes. The number of synonymous substitutions per synonymous site (Ks) was calculated for the orthologous gene pairs using codeml implemented in the PAML program (version 4.9) (Yang, 2007), and the Ks distribution was displayed using R (version 3.6.3). The syntenic gene regions between 93 and 11 and Nipponbare were defined using MCscanX (Wang et al., 2012) with parameters: “-s 5 -m 10 -w 5,” and the tandem duplication genes were obtained from the results.

### Phylogeny Analysis

Protein sequences were generated obtained from six domesticated cultivars (*Oryza sativa indica* cv. 93-11, *O. sativa indica* cv. R498, *O. sativa indica* cv. Minghui63, *O. sativa indica* cv. Zhenshan97, *O. sativa japonica* cv. Nipponbare, and *O. sativa japonica* cv. Kitaake) and 10 wild species (*O. barthii*, *O. brachyantha*, *O. glaberrima*, *O. glumipatula*, *O. longistaminata*, *O. meridionalis*, *O. nivara*, *O. punctata*, *O. rufipogon*, and *Leersia perrieri*)^3^. Then, OrthoFinder (version 2.3.3) (Emms and Kelly, 2019) was used to construct gene families and infer the orthologous and paralogous genes. A total of 1,372 single-copy orthologous genes conserved in these cultivars were found, and multiple alignment of the corresponding protein sequences was performed using Clustal Omega (version 1.2.4) (Sievers et al., 2011) with the default settings. ProtTest (version 3.4.2) (Darriba et al., 2011) was used to estimate the amino acid substitution model for the protein alignment. A phylogenetic tree was constructed using the software RAxML (version 8.0.24) (Stamatakis, 2014) with the parameters “-N 200 -m PROTGAMMAIJTTF -o Leepe,” in which Leepe means the wild species *L. perrieri*.

### Identification of Rapidly Evolving Genes

The orthologous genes of the six species (*O. sativa indica* cv. 93-11, *O. sativa indica* cv. R498, O. *sativa japonica* cv. Nipponbare, *O. sativa japonica* cv. Kitaake, *O. rufipogon*, and *O. nivara*) were identified using OrthoFinder. Multiple protein alignments were performed using Clustal Omega, and the corresponding coding DNA sequence (CDS) alignments were converted using an in-house Perl script. Then, the trimAl (version 1.4) program (Capella-Gutierrez et al., 2009) was used to remove gaps in the CDS alignments. The branch model in PAML with a modified branch-site model, the null model (model = 0), and the alternative model (model = 2) were used to identify the rapidly evolving genes. A likelihood ratio test (LRT) with a df = 1 was performed based on the likelihood values obtained from the two models. Genes with a *P* ≤ 0.05 were considered as rapidly evolving genes in the foreground branch.

### Gene Ontology Enrichment Analysis

GO enrichment analysis and visualization were performed using BiNGO (version 3.04) (Maere et al., 2005) implemented in the Cytoscape (version 3.7.1) (Shannon et al., 2003) software, together with a hypergeometric test. The GO annotation profile of the 93-11 genome was constructed based on the InterPro result, and the ontology file was obtained from the GO website^4^. The GO terms with a *P* ≤ 0.05 were considered significantly enriched.

### Transcript Construction and Gene Expression Analysis

The high-quality RNA-seq reads for each sample were mapped to the 93-11 genome using Hisat2 (version 2.1.0) with the parameters, “–fr –rna-strandness RF,” and the Sam files were converted to a Bam format and sorted using Samtools (version 1.6) (Li et al., 2009) with the default parameters. The transcripts of all samples were constructed using StringTie (version 2.1.2) with the parameter “–rf” and combined using TACO (version 0.7.3) (Niknafs et al., 2017). These transcripts were used to annotate the protein-coding genes. Salmon (version 1.4.0) (Patro et al., 2017) with the parameter “–SS_lib_type RF” was used to map the RNA-seq reads to the transcripts from the 93-11 genome and calculate the expression abundance (transcripts per million (TPM) and read count) of the genes and isoforms.

### Gene Co-expression and Time-Course Analysis

The expression matrix was processed by removing the genes with a TPM < 1 of all samples to analyze the gene co-expression using the weighted correlation network analysis (WGCNA) (Langfelder and Horvath, 2008) package in R. Furthermore, the starch synthesis-related genes and transcription factors with a TPM ≥ 1 were extracted to investigate the gene regulation network related to starch synthesis, and the highly correlated genes (|r| ≥ 0.9 or weight ≥ 0.3) were selected to construct the subsequent regulatory network. Finally, network visualization was performed using Cytoscape (version 3.7.1). Mfuzz (version 2.48.0) (Kumar and E Futschik, 2007) was used to cluster the time-series gene expression data from the 13 stages. The core genes with a membership coefficient value ≥ 0.9 in each cluster were extracted and used for functional analysis.

### Variant Calling

DNA-seq raw sequencing reads were trimmed to remove the low-quality and adaptor sequences using Trimmomatic (version 0.36) (Bolger et al., 2014) with the default parameters. Clean reads were mapped to the 93-11 genome using BWA (version 0.7.17-r1188) (Li and Durbin, 2009). Picard-tools (version 1.119)^5^ were used to remove the duplicate reads from the libraries. GATK (version 3.7) (McKenna et al., 2010) was used to identify the genomic variants: (1) RealignerTargetCreator and IndelRealigner were used to realign the reads to obtain reads with a lower mismatch rate; (2) HaplotypeCaller was used to identify and screen the genome variation (single nucleotide polymorphisms (SNPs and Indels); (3) GenotypeGVCFs was used to combine the variation sites of 14 cultivars.

## Results

### Chromosome Level Genome and Annotation of the 93-11

To construct a high-quality chromosome level 93-11 genome, we used an integrated pipeline described in “Materials and Methods” section using ultra-high depth PacBio long reads (∼196 ×) for assembly and Illumina reads for error correction (Supplementary Table 2). The total length of the assembly was 400 Mb with 281 contigs, and the contig N50 size was 17 Mb (Supplementary Table 3). Furthermore, a total of 24 contigs containing telomere sequences and 26 contigs containing CentO satellite sequences were identified. The assembled contigs were then anchored into 12 chromosomes using 27 Gb Hi-C (chromatin conformation contact) data and via manual correction, and the anchoring rate of the contig sequences was 98.25% (Supplementary Figure 2).

**FIGURE 1:**
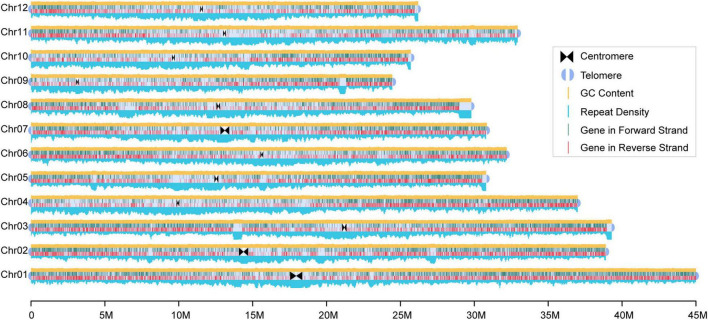
Reference genome of the 93-11. The high-quality chromosome level reference genome of the rice cultivar 93-11 was constructed. The triangle and semicircle represent the centromere and telomere, respectively. Distribution of repetitive sequences and GC content are drawn on the top and bottom of the chromosome with a window size of 100 Kb, respectively. Lines within the chromosomes represent genes, and dark green and red mean locating in the positive and negative strands, respectively.

In total, the final assembled chromosome size was 393 Mb with a scaffold N50 size of 32 Mb (Figure 1 and Supplementary Table 3), which was larger than that of Nipponbare (∼373 Mb). Compared with the previous assembly (Qin et al., 2021), we found good collinearity with fewer collapsed repeat regions between the pseudomolecules (Supplementary Figures 3, 4). In particular, our assembly corrected a potential assembly error of the previous version (Zhang et al., 2018) with a long insertion from chromosome 7 into chromosome 2. The LAI score (Ou et al., 2018), which evaluates the proportion of intact LTR-RT in this 93-11 assembly, was 26.02, indicating that the assembly had relatively high contiguity and quality. Assessment of the completeness of coding genes in the assembly using BUSCO, we observed that approximately 98.3% of the single copy orthologous genes (embryophytes_odb10) were complete (Supplementary Table 4). Additionally, approximately of 99.34% of the Illumina reads, 97.02% of the PacBio long reads, and 95.87% of the RNA-seq reads could be mapped to the genome, further suggesting the completeness of the assembly (Supplementary Table 5). Further evaluation using Merqury revealed that the 93-11 genome had a high-quality score (QV) of 31.55, indicating a high (> 99.9%) assembly accuracy (Supplementary Figure 5). Overall, this is one of the most contiguous and complete assembly of rice genomes published to date.

This 93-11 assembly contained 190 Mb repetitive sequences, accounting for 48.28% of the genome, including 14.41% of DNA transposons, 28.11% of retrotransposons, and 1.12% of simple repeats. The total repeat length of the 93-11 genome was greater than that in the Nipponbare genome (Supplementary Table 6).

Protein-coding genes in the 93-11 genome were annotated using multi-evidence integrating strategy with manual correction based on pieces of evidence obtained from the transcriptome and Nipponbare gene models. A total of 40,345 genes were identified with an average length of 2,895 bp (Supplementary Table 7). Of all the annotated genes, 90.18% were aligned to the Nr database, 88.22% had functional annotations from the InterPro database, 55.07% (22,218) contained Pfam domains, and 42.28% (17,059) and 21.20% (8,555) were assigned to the GO terms and KEGG Orthology identifiers, respectively (Supplementary Table 8). Moreover, 1,616 transcription factors were detected and most of them belonged to the bHLH family (204).

### The Genomes of 93-11 and Nipponbare Are Highly Similar, and Comparison of Gene Sets Showed Cultivar Specific Genes Accounting for Various Functions

To compare the 93-11 genome and Nipponbare genomes, we first cross-mapped the Illumina reads to each assembly. Approximately 98.21 and 97.88% of the reads were mapped to the 93-11 and Nipponbare genomes, respectively. Collinearity analysis between the 93-11 and Nipponbare genomes showed that approximately 91.12% of the 93-11 genome sequences had one-to-one syntenic blocks with the Nipponbare genome (nucleotide identity of 97.21%). These results confirmed that the two genomes were very similar.

**FIGURE 2:**
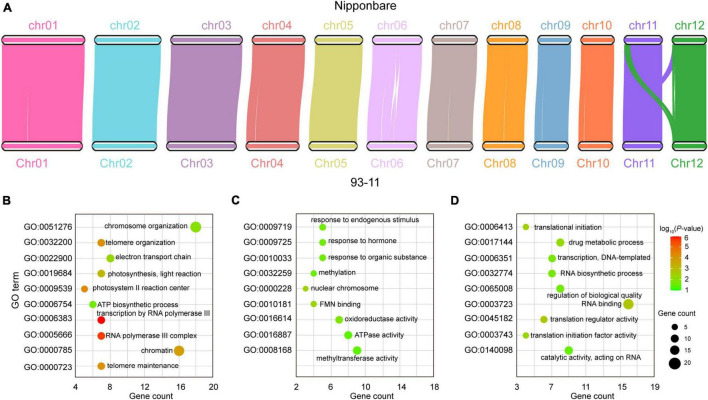
Comparative genomic analysis of 93-11 and Nipponbare. **(A)** Collinearity between the 93-11 and Nipponbare genomes. There are 27,729 homologous genes in the collinear regions, indicating that the two genomes have a high degree of collinearity. **(B)** Gene ontology (GO) enrichment of the genes in non-syntenic regions. **(C)** GO enrichment of the rapidly evolving genes in the 93-11 genome. **(D)** GO enrichment of the rapidly evolving genes in the Nipponbare genome. These results indicate the species specificity of gene evolution after species differentiation. The color scale indicates the *P-*value, and the size of the circle indicates the number of genes.

A total of 32,961 genes in the 93-11 genome were homologous to those in Nipponbare, of which 27,729 genes were in the syntenic regions and 5,232 genes were in the non-syntenic regions (Figure 2A). Comparative genomics analysis showed that those genes in the syntenic region were more conserved, while the non-syntenic genes had higher evolution rates (Supplementary Figure 6). The non-syntenic genes was enriched in the following GO terms: telomere maintenance, telomere organization, and chromosome organization (Figure 2B and Supplementary Table 9), suggesting diversity in chromatin variation between the 93-11 and Nipponbare genome, which still need to be further validated with improved Nipponbare due to incompleteness of current Nipponbare genome. We further checked unaligned proteins specific to both genomes, and identified 1,026 genes and 743 in the 9,311 and Nipponbare genomes, respectively, most of which were hypothetical proteins.

Rapid evolution results in the generation of novel genes that enables better adaptation to the environment (Crow et al., 2006). We identified 498 and 481 rapidly evolving genes in the 93-11 and Nipponbare genome, respectively. Interestingly, the functions of these two gene sets were significantly different. The rapidly evolving genes in the 93-11 genome were enriched in functions related to response to endogenous stimulus, response to organic substance, methylation, and cellular nitrogen compound metabolic process (Figure 2C and Supplementary Table 10), whereas those in the Nipponbare genome were related to the organic substance biosynthetic process, translational initiation, and regulation of biological quality (Figure 2D and Supplementary Table 11). These genes may account for the high disease-resistance character of the 93-11 rice and the good cooking quality of Nipponbare rice.

### Phylogeny Analysis Indicates the Different Origins of *Indica* and *Japonica*

**FIGURE 3:**
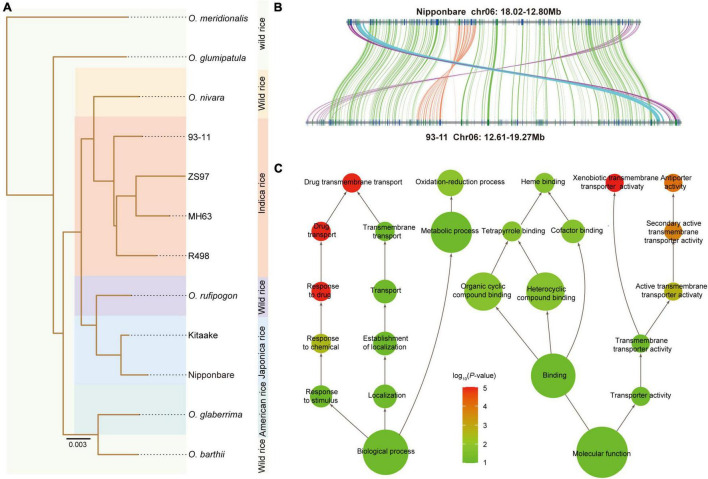
Chromosome inversion events. **(A)** Phylogenic tree of cultivars and wild species of *Oryza*. **(B)** Collinearity between 93-11 and Nipponbare shows the presence of a paracentric inversion (orange lines) within the pericentric inversion (green lines). Additionally, another paracentric inversion (cyan lines) next to the pericentric inversion was also identified. **(C)** Functional enrichment of the genes in the inversion region. The color scale indicates the *P*-value, and the size of the circle indicates the number of genes.

Despite the high synteny and sequence similarity between the 93-11 and Nipponbare genome, the phylogenetic tree revealed that the 93-11 (representing *indica*) and Nipponbare (representing *japonica*) were clustered with two different wild species, *O. nivara* and *O. rufipogon* (Figure 3A and Supplementary Figure 7), which was agreed with the *Ks* results (Supplementary Figure 8). Nucleotide similarity analysis showed that the sequence similarity between 93-11 and *O. nivara* and between Nipponbare and *O. rufipogon* was 98.07 and 98.27%, respectively, which are both higher than that between 93-11 and Nipponbare (97.20%) (Supplementary Table 12). In conclusion, it is highly likely that the rice *japonica* and *indica* groups may arise separately from the progenitor *O. rufipogon* and *O. nivara*, rather than differentiating into subspecies, which confirms the results reported in a previous studies (Choi et al., 2017; Stein et al., 2018). This suggests that mapping sequencing data from the rice *indica* group to the Nipponbare genome might be incorrect. Therefore, it is always a good idea to map sequencing data of the *indica* cultivars to the 93-11 genome and vice versa.

### Comparative Analysis Showed an Inversion Which Nestled Another Inversion Region

To discover the structural variations (SVs) that potentially shaped the 93-11 cultivar, compared with the Nipponbare genome, we identified 8,188 large fragment insertions (≥ 1 Kb) and 9,045 inversions in in the 93-11 genome. The insertions and inversions account for 8.73% (34 Mb) and 9.80% (38 Mb) of the 93-11 genome, respectively. Notably, there was one remarkable pericentric inversion that was found to be specific for 93-11, which was located at 12994554–18810736 on chromosome 6 (corresponding to chromosome 6:13119682–17632495 in Nipponbare) (Figure 3B and Supplementary Figure 9). Interestingly, we found a nestled inversion region of 700 Kb within the pericentric region, located at 14563302−15266093 in the short arm of chromosome 6 (corresponding to chromosome 6: 15702521–16205397 in Nipponbare) (Figure 3B). It looks like a secondary inversion, but it is not clear whether this nestled inversion is reserved when the inter-arm inversion occurs or if it is inverted after the inter-arm inversion occurs. In addition, another paracentric inversion (93-11: 18812083–19050675; Nipponbare: 17659075–17900016) next to the remarkable inversion was identified. These inversions in the 93-11 genome were also confirmed by PacBio read coverage visualization (Supplementary Figure 10).

The inversion regions carried 367 genes, of which 187 were homologous to those of Nipponbare (Supplementary Table 13), and 224 were expressed during seed development. Genes encoding P450, NB-ARC, and multi-antimicrobial extrusion proteins were identified, which are related to disease resistance and seed development function (Supplementary Table 13). Functional enrichment was mainly included in response to stimulus/chemical/drug, transmembrane transporter activity, xenobiotic transmembrane transporter activity, oxidation-reduction process, organic cyclic compound binding, and cofactor binding (Figure 3C and Supplementary Table 14), which demonstrates the potential high disease resistance character of 93-11. Nine transcription factors were also found in this region, including one bHLH, one NAC, one ZF-HD, one CPP, one G2-like, one MIKC_MADS, one M-type_MADS, and two HB-other. In addition, the Ks result indicated that the genes in the inversion regions were evolving significantly faster than the other genes on chromosome 6 (Supplementary Figure 11).

Overall, these results demonstrate that large SVs may significantly contribute to cultivar formation and may facilitate the rapid adaptation or domestication of cultivars. The identified SVs could also be used as molecular markers to distinguish between *indica* and *japonica* for genetic traceability.

### Continuous Sampling Provides Time-Course Insight on the Transcriptomic Pattern During Seed Development

The time-course RNA sequencing approach provides an opportunity to better evaluate gene expression patterns with sample collection in parallel over time (Spies and Ciaudo, 2015; Thomas et al., 2020). We collected samples at 13 stages from 4 to 16 DAP to understand the seed developmental processes of rice using integrated multiple methods based on TPM values of the 13 stages, including Pearson correlation, hierarchical clustering (HC), WGCNA, and transcriptome-wide time series expression analysis. The results demonstrated that the number of expressed genes (EGs) and stage-specific expressed genes (SSEGs) increased from 4 to 5 DAP and decreased from 5 to 16 DAP. At 5 DAP, the most EGs and SSEGs were observed (19,255 and 441, respectively) (Supplementary Table 15 and Supplementary Figure 12). The functions of the stage-specific highly expressed genes (SSHEGs) and SSEGs at 5 DAP were mainly involved in defense response, response to stress, carbohydrate metabolic process, cell wall biogenesis, catalytic activity, hydrolase activity, peptidase regulator activity, etc. (Supplementary Table 16). All these results indicate that the rice seed development had entered an active state from this stage; the content of the seed had been synthesized quickly, and the resistance to external biological stimuli was enhanced.

**FIGURE 4:**
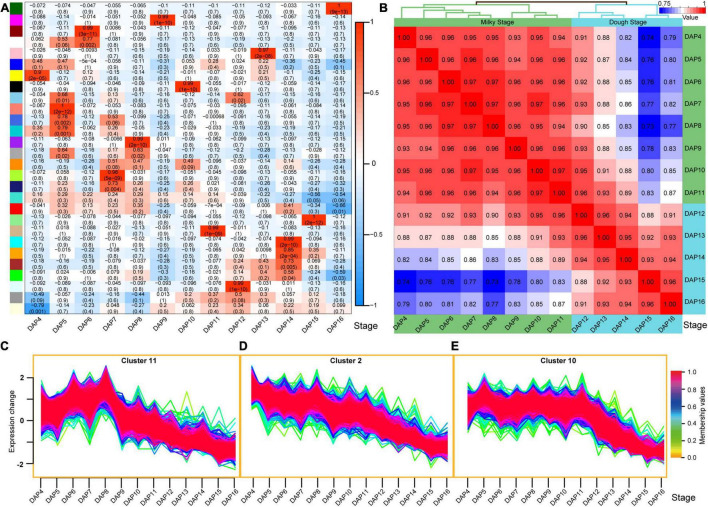
Gene expression patterns during seed development. **(A)** Correlation between the developmental stages and expression modules. A total of 28 modules were identified by the weighted correlation network analysis (WGCNA), of which 13 modules are highly correlated with 13 developmental stages (*r* > 0.9). **(B)** Pearson correlation of the 13 stages based on all of the expressed genes. These stages are divided into two groups, which corresponded to the milky and dough period. **(C–E)** Typical gene expression clusters. Cluster 11 indicates high expression in the first five stages and low expression in the subsequent stages **(C)**; cluster 2 means that gene expression level was decreasing during seed development (from DAP4 to DAP16) **(D)**; cluster 10 indicates high expression in the milky period and low expression in the dough period **(E)**.

A total of 28 expression modules (except for the gray module) were identified using WGCNA, and 13 modules were highly correlated with the 13 stages (*r* > 0.9) (Figure 4A). The analysis was consistent with the results from the transcriptome-wide time-series expression analysis (Supplementary Figure 13), which indicates that the gene expression was stage-specific, and the expression pattern of the core genes in each module could be used as a marker for each stage. Hierarchical clustering analysis results showed two main groups, corresponding to the milky and dough stages of rice seed development (Figure 4B and Supplementary Figure 14), which were also identified based on their morphology. Each main group was further divided into two sub-groups, corresponding to the early and late periods of their respective stages.

Functional analysis of the highly EGs in the first five stages (early stage of milky stage) (Figure 4C) showed that these genes were mainly involved in biosynthetic processes, genes expression, translation, binding, enzymatic activity (such as hydrolase, ATPase, ligase, oxidoreductase, pyrophosphatase, etc.), and regulation of translation (Supplementary Table 17). This indicated that the material synthesis in the seed was active during these stages, and the content began to accumulate rapidly. We also found that 1,004 genes related to the above-mentioned biological processes (Supplementary Table 18) showed the expression levels in the late stages (Figure 4D), which further indicates that these biological processes, such as content synthesis, slowed down in the late stages of seed development, leading to a gradual accumulation of the content in the seeds.

The genes in cluster 10 showed high expression levels during the milky stage but low expression levels during the dough stage (Figure 4E). Functional enrichment analysis revealed that these genes were highly associated with vesicle-mediated transport, intracellular transport and localization, cellular nitrogen compound metabolism, cytoskeleton organization and regulation, protein-containing complex, organelle, cytoskeleton, binding, intramolecular transferase activity, peptidase activity, structural constituent of cytoskeleton, etc. (Supplementary Table 19). These results indicate that the stage-specific genes expressed at the milky stage may be highly related to the formation of macromolecular structures during seed development. The expression pattern of these genes could be used as a marker of the seed development transition from the milky to the dough stages.

### Gene Regulation Network Related to Starch Synthesis During Endosperm Genesis

**FIGURE 5:**
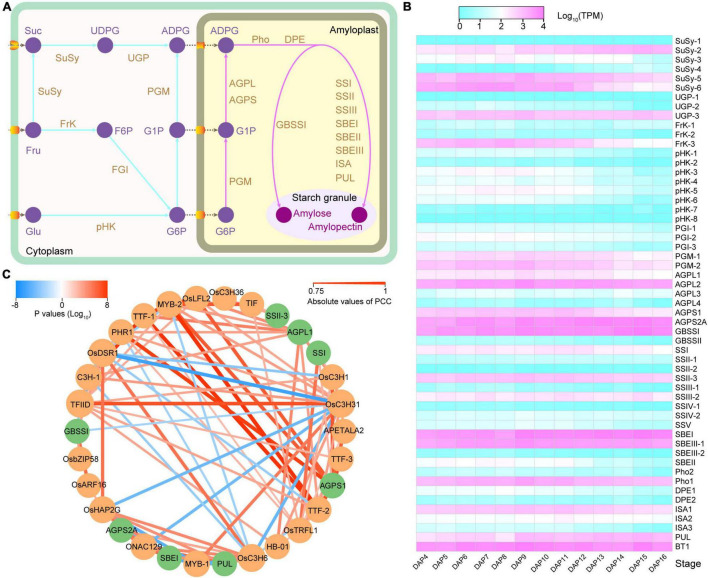
Gene expression and regulation network of starch synthesis-related gene. **(A)** Starch synthesis pathway in rice endosperm. **(B)** Starch synthesis-related gene expression profile. **(C)** Co-expression network of 8 starch synthesis-related genes (SSRGs) and 22 transcription factors (TFs). The green and orange colors represent SSRGs and TFs, respectively. The line color indicates the *P*-value, and the red and blue shades represent the positive regulation and negative regulation, respectively. The width of the line represents the correlation; the wider the line, the higher is the correlation.

Starch is synthesized in amyloplasts from its initial substrates, glucose 1-phosphate (G1P) and glucose 6-phosphate (G6P). G1P and G6P are imported into an amyloplast and synthesized to amylose and amylopectin by several enzymes playing orchestrated roles, including phosphoglucomutase (PGM), ADP-glucose pyrophosphorylase (AGP), granule-bound starch synthase (GBSS), soluble starch synthase (SS), starch branch enzyme (SBE), starch debranching enzyme (DBE), starch phosphorylase (Pho), and dismutase (DPE) (Figure 5A). In total, 32 starch synthesis-related genes (SSRGs) were annotated in the 93-11 genome (Figure 5B and Supplementary Table 20). Gene expression analysis demonstrated that there were significant differences in the expression levels of the SSRGs, as well as in the expression patterns during seed development.

We further analyzed the co-expression network of the SSRGs and transcription factors (TFs) using WGCNA. A total of seven modules containing SSRGs were identified, and highly correlated connections (|r| ≥ 0.9 or weight ≥ 0.3) were selected to construct the subsequent regulatory network (Figure 5C and Supplementary Figure 15). Eight SSRGs and 54 TFs participate in the network. Among the TFs, 22 of them were directly involved in regulating the expression of SSRGs, of which the bZIP transcription factor R9311C07g25353 (corresponding to OsbZIP58) regulated starch synthesis in rice endosperm (Supplementary Tables 21, 22; Wang et al., 2013). The network showed that these TFs belonged mainly to the C3H and MYB families, including 5 and 4 genes, respectively. In addition to OsbZIP58, R9311C01g03921 (OsDSR1), R9311C04g17870 (OsC3H31), R9311C09g31905, and R9311C06g22141 (OsARF16) may also be involved in regulating the expression of *Wx*. R9311C09g31905 is a transcription initiation factor that showed a negative regulation (*r* = −0.8439) with *Wx* expression (Supplementary Table 22). In addition, three transcription termination factors (R9311C06g22381, R9311C06g22379, and R9311C11g36090) were detected in the network (Supplementary Table 21). Overall, the network indicated that these TFs may play a vital role as potential regulators of SSRGs expression and starch synthesis.

### Variations in Alternative Splicing Reveals Isoform Preferences in Amylose Synthesis-Related Genes

Alternative splicing is an important post-transcriptional regulatory mechanism that increases proteome diversity by altering the mRNAs. Interestingly, based on the endosperm transcriptome data, we noticed a special type of alternative splicing event induced by splice acceptor “drift” at the acceptor splice site (ASS) of the first intron of three SSRGs, *BT1* (*R9311C02g06322*), *AGPL2* (*R9311C01g02861*), and *Wx* (*R9311C06g21642*) (Supplementary Figure 16). Briefly, the splice site variation was due to multiple occurrences of the AG sequences in the 3′-ends of the first intron. In *BT1* and *AGPL2*, the sequence of the 3′-end was TAGCAGCAG and TAGTTGCAG, producing three and two acceptor splice sites, respectively. Two ASSs in *Wx* were generated using the CAGTGCAG. In summary, adjacent AGs can easily form alternative splice sites in the pre-mRNA.

**FIGURE 6:**
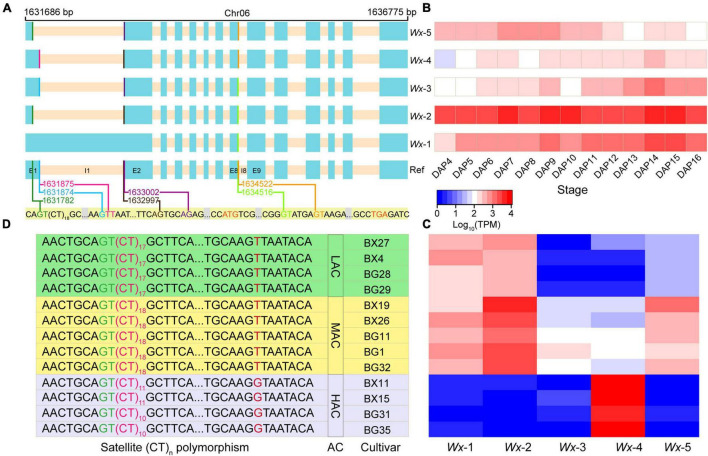
Alternative splicing and satellite (CT)_n_ polymorphism in the *Wx.* **(A)** Three donor splice sites (forestgreen, deepskyblue, and deeppink lines) and two acceptor splice sites (brown and darkviolet lines) in the first intron were identified. Two donor splice sites (lawngreen and orange lines) in the eighth exon were also detected, which related to the trinucleotide CAG. So, the pre-mRNA of *Wx* can produce five transcripts (*Wx*-1, *Wx*-2, *Wx*-3, *Wx*-4, and *Wx*-5). The cyan and bisque rectangles represent the exon and intron, respectively. **(B)** Expression levels of the five isoforms at different stages of seed development. **(C)** Expression levels of the five transcripts in the rice cultivars. **(D)** Satellite (CT)_n_ polymorphism in the rice population. The polymorphism is closely related to the alternative splicing in the *Wx* and amylose content, which indicates the potential regulation of the splicing and amylose content in the rice cultivars. BX and BG represent the *inidca* and *japonica* cultivars, respectively. AC represents amylose content, while LAC, MAC, and HAC represent low, medium, and high amylose content, respectively.

In addition to the ASS in the first intron, three donor splice sites (DSSs) in the first intron and two alternative DSSs in the eighth intron were identified, located at 1631782, 1631874, 1631875, 1634516, and 1634522 in chromosome 6, respectively (Figure 6A). These alternative splice sites resulted in the formation of five isoforms (*Wx*-1/*Wx*-2/*Wx*-3/*Wx*-4/*Wx*-5) of the *Wx* gene in the 93-11 cultivar (Figure 6A). *Wx*-1 and *Wx*-4 corresponded to *Os06t0133000*-02 and *Os06t0133000*-01 of Nipponbare, respectively. It is known that an SNP (T/G) at the 5′ end of the first intron of the *Wx*, which is located exactly at the splice donor site of the first intron, determines the retention of the first intron, thus forming two isoforms *Os06t0133000*-02 (T: intron retention) and *Os06t0133000*-01 (G: intron skipping). In the 93-11 genome, the SNP (chromosome 6:1631875) was of the T type, which is the same as that of Nipponbare; however, surprisingly, both isoforms could be detected in the transcriptome. This indicates that there are still some pre-mRNAs that could use this splicing site to produce mature mRNA.

As reported in a previous study (Frances et al., 1998), the proper splicing of the first intron of the *Wx* is critical for activating the GBSSI enzyme. Two cryptic DSSs (1631782 and 1631874) were detected in the *Wx* of 93-11 to form *Wx*-2, *Wx*-3, and *Wx*-5, which excluded the first intron. Interestingly, the expression levels and patterns of these five transcripts were significantly different (Figure 6B and Supplementary Table 23). Notably, the expression level of the *Wx*-2 isoform was much higher than those of the other isoforms. This showed that the *Wx*-2 isoform was the preferred in 93-11 and might be the main source of active GBSSI enzyme during seed development.

### The Isoform Preference Is Closely Related With the (CT)_n_ Microsatellite Polymorphism in the *Waxy*

A previous study (Bao et al., 2002) has reported that there was a (CT)_n_ polymorphism downstream of I1-DSS in the rice population. To investigate if the (CT)_n_ variation results in a *Wx* preference by impacting the ASS selection of the first intron, we further expanded the transcriptome analysis to 13 rice cultivars (Supplementary Table 1). Gene expression analysis showed that *Wx*-2 was highly expressed only in the medium amylose content (AC) cultivars (Figure 6C). The genotyping of (CT)_n_ demonstrated that the length of (CT)_n_ is closely related to AC in rice cultivars (Figure 6D): (CT)_17_, (CT)_18_ and (CT)_11/12_ were present in low, medium, and high AC cultivars, respectively. Interestingly, in comparison with the low AC cultivars, a slight extension of (CT)_n_ from *n* = 17 to *n* = 18 could activate the expression of the *Wx-2* isoform, standing out from the low robust *Wx-1.* This result suggests that in addition to splice site variations, (CT)_n_ may also play an important role in the splicing efficiency and isoform preference in rice cultivars.

In addition, we investigated the potential regulators involved in pre-mRNA splicing of the *Wx* using Pearson correlation and WGCNA based on all the expressed transcripts at the 13 stages. A total of 52 identified genes had a high correlation coefficient (|r| > 0.8) with any of the five isoforms of the *Wx*. These genes included 14 genes with an RNA recognition motif domain (PF00076) and six splicing factors (Supplementary Table 24). Two Ser/Arg-rich proteins, R9311C04g14651 (Os-RSp29) and R9311C02g08144 (Os-RSZ23) and a dull endosperm1 R9311C10g34727 (Du1) were reported to be involved in the splicing of the *Wx* pre-mRNA. The analysis revealed that these genes may be potential regulatory factors for the alternative splicing of the *Wx* pre-mRNA.

## Discussion

To date, over 150 rice genome assemblies have been deposited in NCBI, of which 44 are chromosome-level assemblies and one is a complete genome assembly (Benson et al., 2013). Over the past few decades, a complete telomere to telomere (T2T) assembly has always been the final goal of all genome projects. As sequencing technology evolves and its costs are being reduced, we are now closer to achieving this goal than ever before. Currently, the biggest challenge in constructing a T2T complete genome is the complexity of the centromeres and the highly duplicated transposable elements (Li et al., 2021; Rhie et al., 2021). That is mainly because the high read length and the low error rate do not converge with each other. In this study, we attempted to use long reads with an ultra-depth (∼196 ×) for assembly, with a much lower corrected error rate while maintaining a relatively high read length. The results from this study could help solve the problems associated with the low-complexity regions, such as the telomeres and centromeres. The results showed that this strategy outperformed those used for previous assemblies as it yielded higher continuity. Using Hi-C scaffolding, short-read polishing, and manual curation, we elevated the reference genome of 93-11 to a higher quality level. This assembly not only provides robust data support for genome variation analysis, but also provides a valuable reference for applying ultra-deep coverage long-read assembly for further improvement of other complex plant genomes.

Owing to the lack of sufficient transcriptome data and the false positive error of annotation tools, manual checking is an efficient way to strengthen the automatic annotation result. We manually confirmed around 3,000 doubtful genes and revised them, which provided a reliable annotation file compared with the reference annotation sets. Orthologous analysis between the 93-11 and Nipponbare genomes yielded many orphan genes (7,383 in 93-11 and 3,750 in Nipponbare). However, when these orphan genes were cross mapped to the 93-11 and Nipponbare genomes, the number of species-specific genes was significantly reduced (1,026 in 93-11 and 743 in Nipponbare). This phenomenon maybe due to differences between the cultivars, a lack of a phased (diploid) genome, or errors in gene annotation, and further in-depth research is required. Although the two genomes have high similarities, there are significant differences in the number of genes and genome structure between the two cultivars. Differences between the two genomes make it inappropriate to use Nipponbare as a reference genome to explore *indica* cultivars. Therefore, it is necessary to construct a high quality *indica* rice reference genome or pan-genome.

A previous study showed that the *Wx* and *SSIIa* seem to be the main trait loci for the domestication in rice (Kharabian-Masouleh et al., 2012). In this study, we found that the complexity of the *Wx* isoforms and their preferences could be used to distinguish the AC of cultivars. The variation in the *Wx* of rice offers several options for molecular breeding programs. Previous studies have shown that the polymorphism of simple repeat (CT)_n_ occurring in the 5′UTR region of genes could influence the promoter activity and gene expression in plants. In this study, we found that even tiny changes in microsatellite (CT)_n_ motif could influence the isoform preference of the *Wx* and resulted in an apparent change in AC, which indicates the sophisticated mechanism behind splicing factors and splicing-related proteins that mediates alternative splicing.

## Conclusion

In conclusion, our study demonstrates that the near-complete genome assembly is feasible to efficiently decipher multi-omics data. Moreover, the reference genome of the rice cultivar 93-11 could be used as a model to study the AC-related traits and the complex genome variations between *indica* and *japonica*, which may be helpful for the future breeding programs.

## Data Availability Statement

The datasets presented in this study can be found in online repositories. The names of the repository/repositories and accession number(s) can be found below: http://bigd.big.ac.cn/gsa, GSA: CRA00469 and CRA005384, https://bigd.big.ac.cn/gwh, GWH: GWHBEBR00000000.

## Author Contributions

SeW, PC, SH, and SYW conceived this study and wrote the manuscript. SeW, SG, and SYW designed the experiments, collected the samples, and performed the analyses. JN and JX collected the data and partially performed the analysis. XT, XB, YS, SL, QZ, JG, WL, and QL assisted in sample collection. All authors contributed to the article and approved the submitted version.

## Conflict of Interest

The authors declare that the research was conducted in the absence of any commercial or financial relationships that could be construed as a potential conflict of interest.

## Publisher’s Note

All claims expressed in this article are solely those of the authors and do not necessarily represent those of their affiliated organizations, or those of the publisher, the editors and the reviewers. Any product that may be evaluated in this article, or claim that may be made by its manufacturer, is not guaranteed or endorsed by the publisher.

## Footnotes

1
https://genomethreader.org/

2
https://www.genome.jp/kegg/

3
https://plants.ensembl.org/index.html

4
http://geneontology.org/

5
http://picard.sourceforge.net

## Abbreviations

AC: amylose content
DAP: day after pollination
SMRT: single molecule real-time
*Wx*: *waxy*
TRs: tandem repeats
JBAT: Juicebox Assembly Tool
BUSCO: Benchmarking Universal Single-copy Orthologs
TE: transposable element
EDTA: Extensive *de novo* TE Annotator
ORFs: open reading frames
BLASTP: protein-protein basic local alignment search tool
NR: non-redundant protein database
KEGG: Kyoto Encyclopedia of Genes and Genomes
GO: gene ontology
Ks: the number of synonymous substitutions per synonymous site
CDS: coding DNA sequence
PAML: phylogenetic analysis by maximum likelihood
LRT: likelihood ratio test
TPM: transcripts per million
WGCNA: weighted correlation network analysis
SNPs: single nucleotide polymorphisms
EGs: expressed genes
SSEGs: stage-specific expressed genes
SSHEGs: stage-specific highly expressed genes
G1P: glucose 1-phosphate
G6P: glucose 6-phosphate
PGM: phosphoglucomutase
AGP: ADP-glucose pyrophosphorylase
GBSS: granule-bound starch synthase
SS: soluble starch synthase
SBE: starch branch enzyme
DBE: starch debranching enzyme
Pho: starch phosphorylase
DPE: dismutase
SSRGs: starch synthesis-related genes
TFs: transcription factors
ASS: acceptor splice site
DSSs: donor splice sites
I1: the first intron
I7: the seventh intron
Du1: dull endosperm1
T2T: telomeres to telomere.
