# Supplementary material for: Improved 93-11 Genome and Time-Course Transcriptome Expand Resources for Rice Genomics
Source: Front Plant Sci. 2022 Jan 21;12:769700. doi: 10.3389/fpls.2021.769700 (PMC8813773; doi:10.3389/fpls.2021.769700)
Supplement: Supplementary file 1 [file Data_Sheet_1.ZIP › Supplementary Material/Supplementary Figures.pdf]

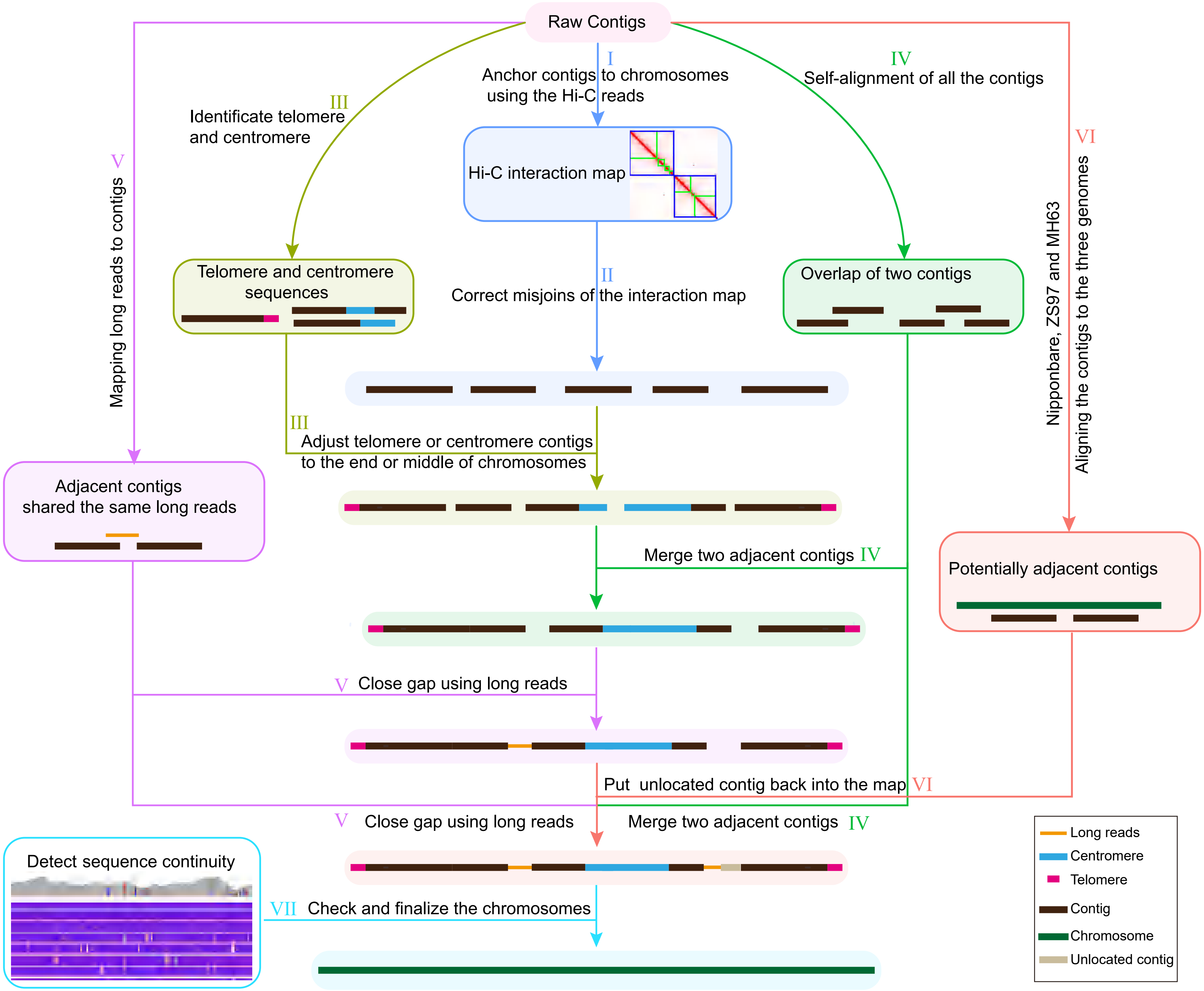

Supplementary Figure 1. Pipeline of the improved assembly of 93-11. (I) All the contigs were anchored to chromosomes using the Hi-C reads to construct interaction map. (II) Misjoins of the interaction map were corrected. (III) Telomere and centromere sequences (pink and blue) were identified, and adjusted to the end or middle of chromosomes. (IV) Self-alignment of all the contigs was performed to merge two adjacent contigs. (V) Gaps between adjacent contigs were closed using the long reads. (VI) Unlocated contig (light brown) was put back into the map, and step IV and V were performed again. (VII) Long reads were aligned to the polished contigs to detect sequence continuity, we manually checked the errors and finalized the chromosome.

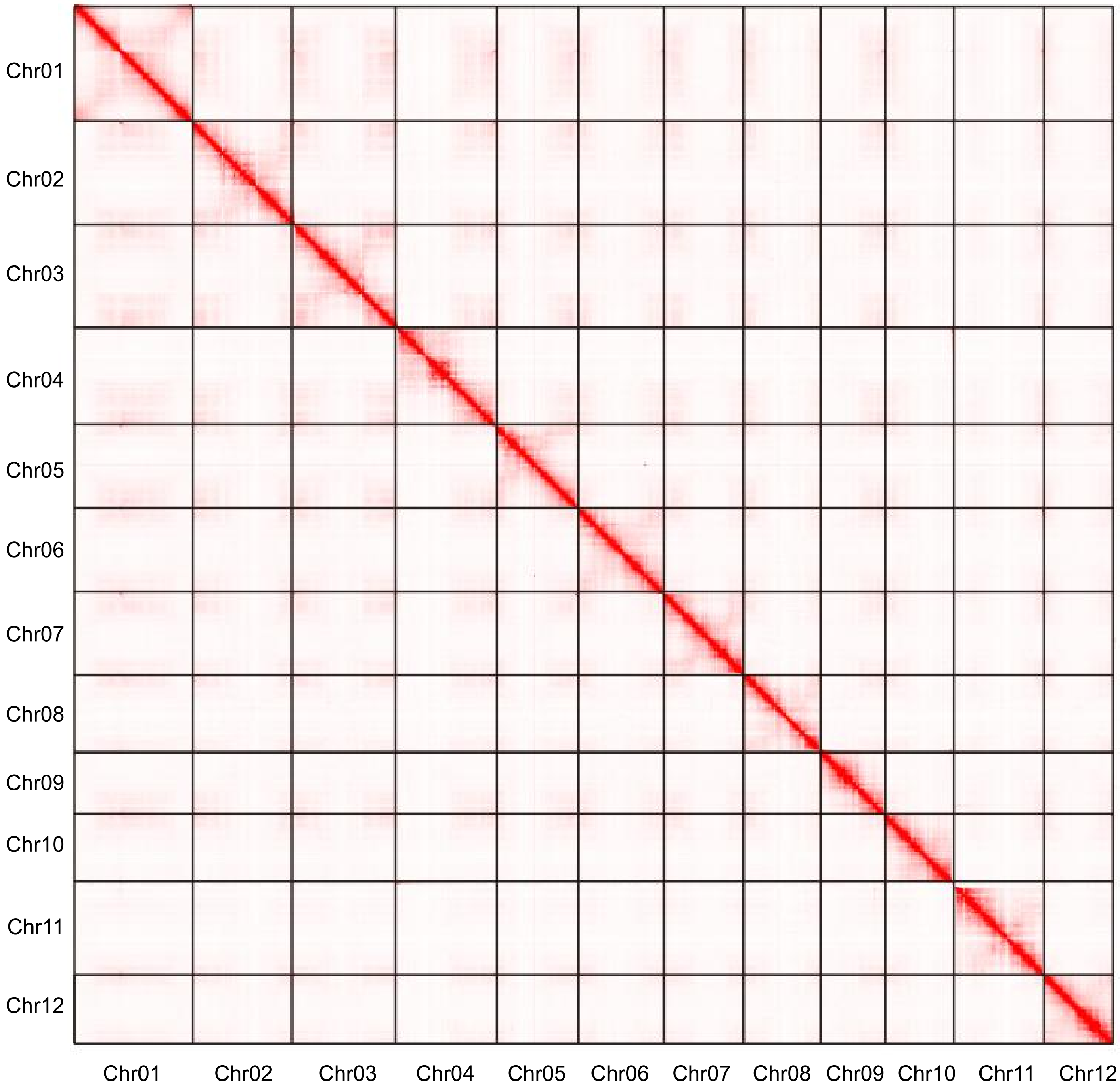

Supplementary Figure 2. Hi-C interaction map of the 93-11 genome with 12 chromosome-level pseudomolecules.

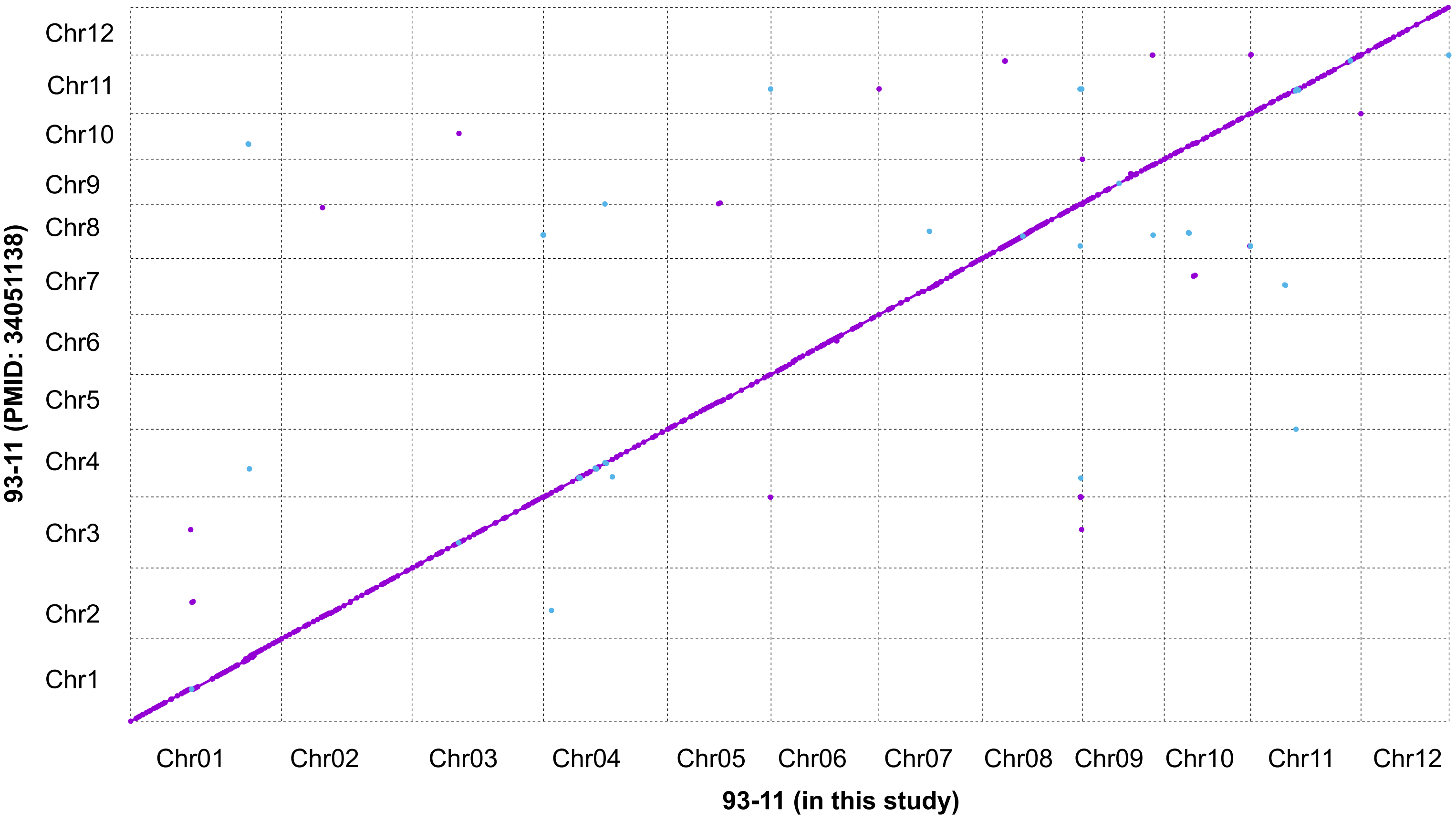

Supplementary Figure 3. Synteny comparison between 93-11 and the previous version (PMID: 34051138). The high synteny indicates less collapsed repeat region between the two assemblies.

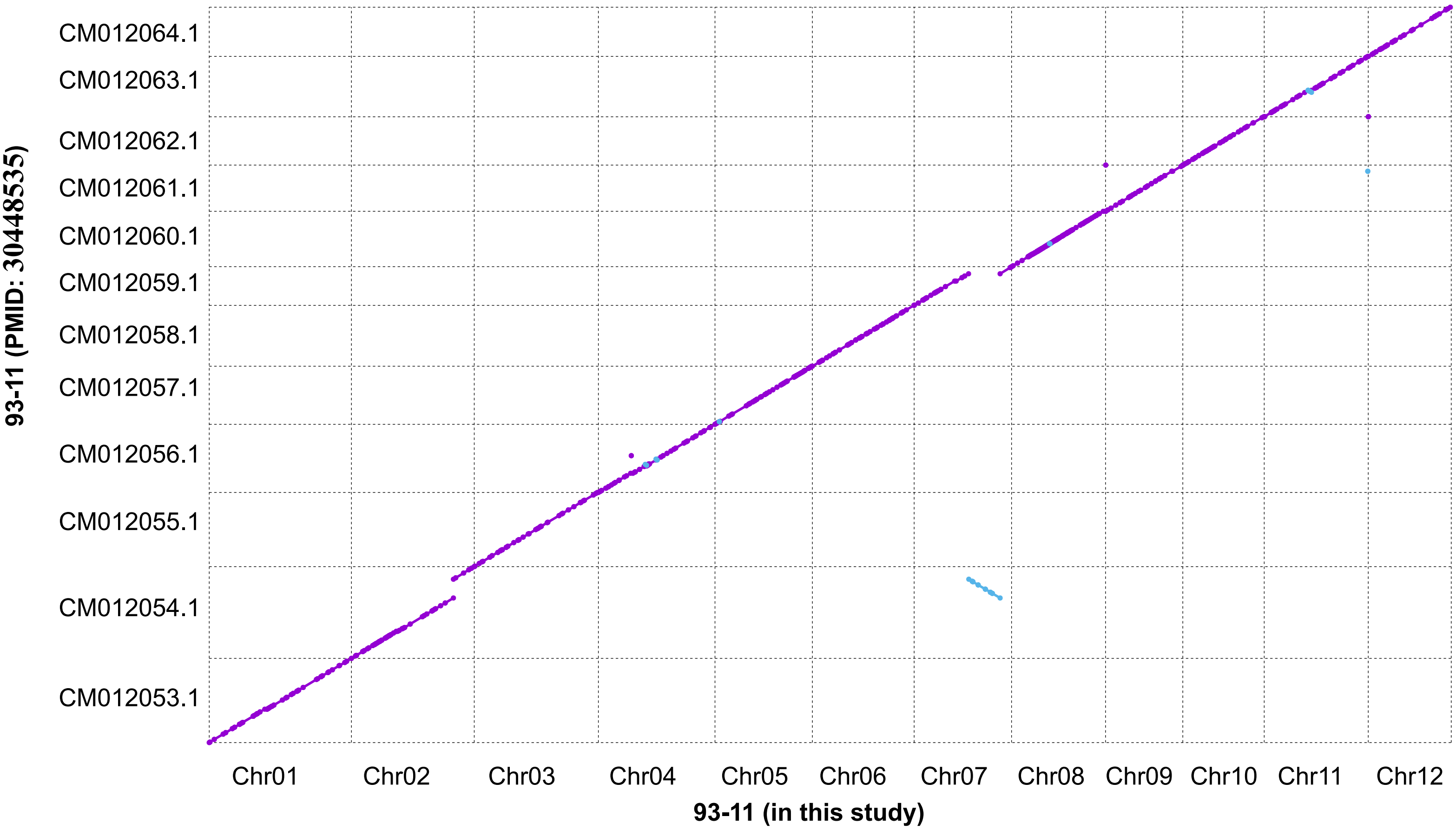

Supplementary Figure 4. Synteny comparison between 93-11 and the previous version (PMID: 30448535). The high synteny indicates less collapsed repeat region between the two assemblies.

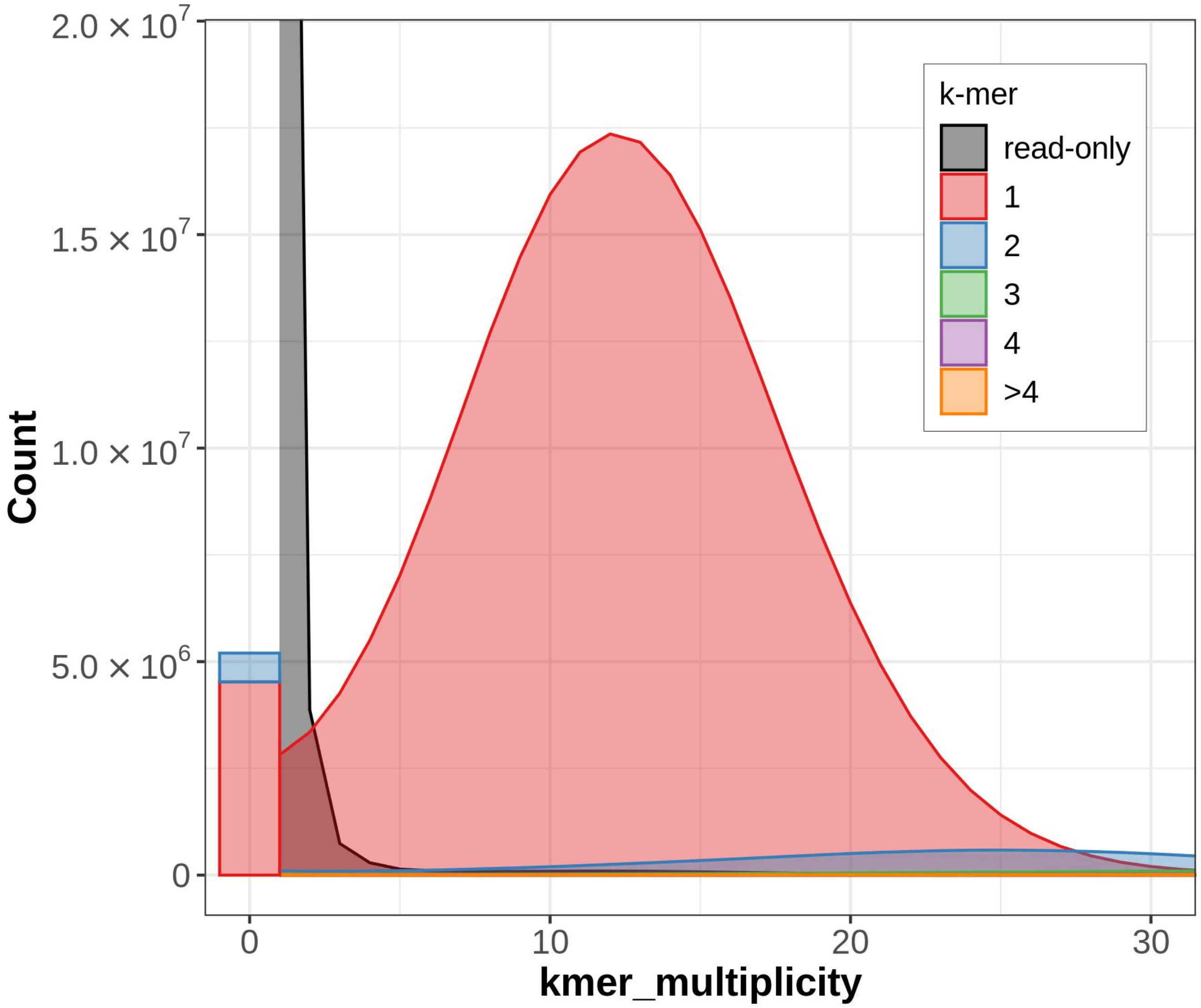

Supplementary Figure 5. K-mers evaluation of genome assembly completeness by merqury. Color means copy numbers detection in the assembly.

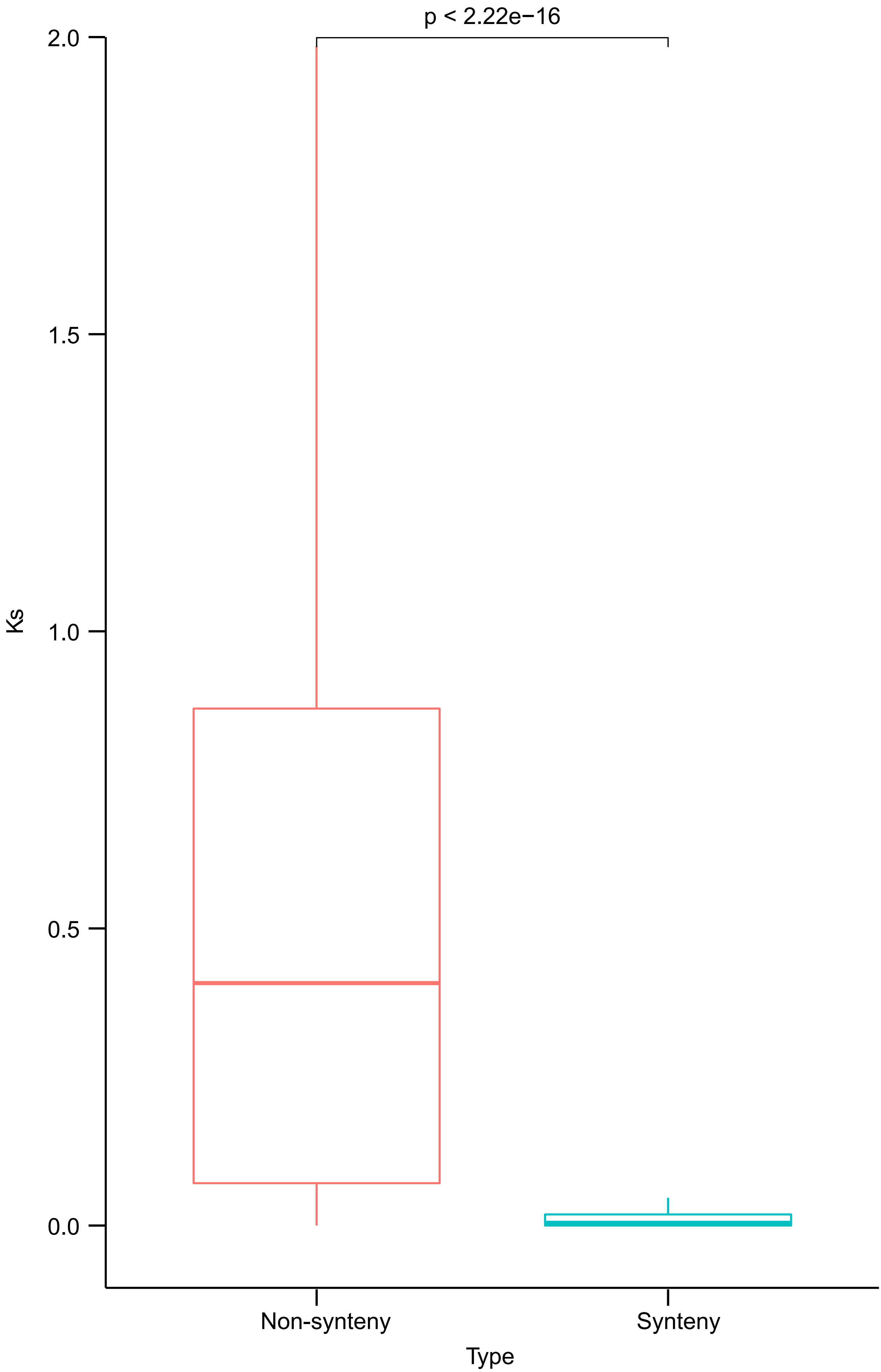

Supplementary Figure 6. Ks value comparison of homologous genes in the synteny and non-synteny region. The non-synteny genes have a higher evolution rate than synteny genes (p value <2.22e-16).

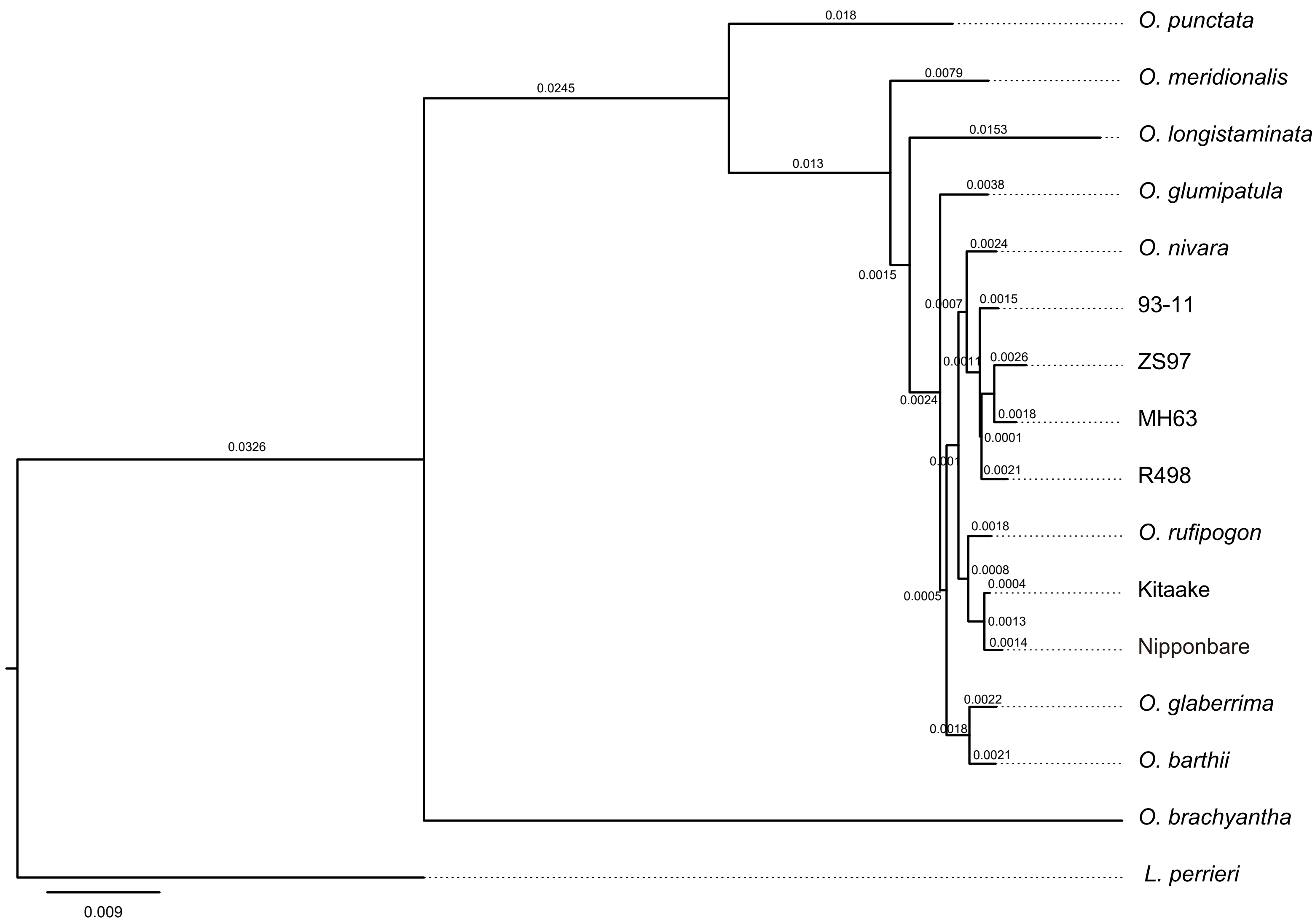

Supplementary Figure 7. Phylogenic reconstruction of cultivars and wild species of Oryza. The phylogenetic tree was constructed using concatenated amino acid sequences for 1372 single-copy genes present in all genomes through maximum likelihood analysis. The numbers on each branch indicate the distance.

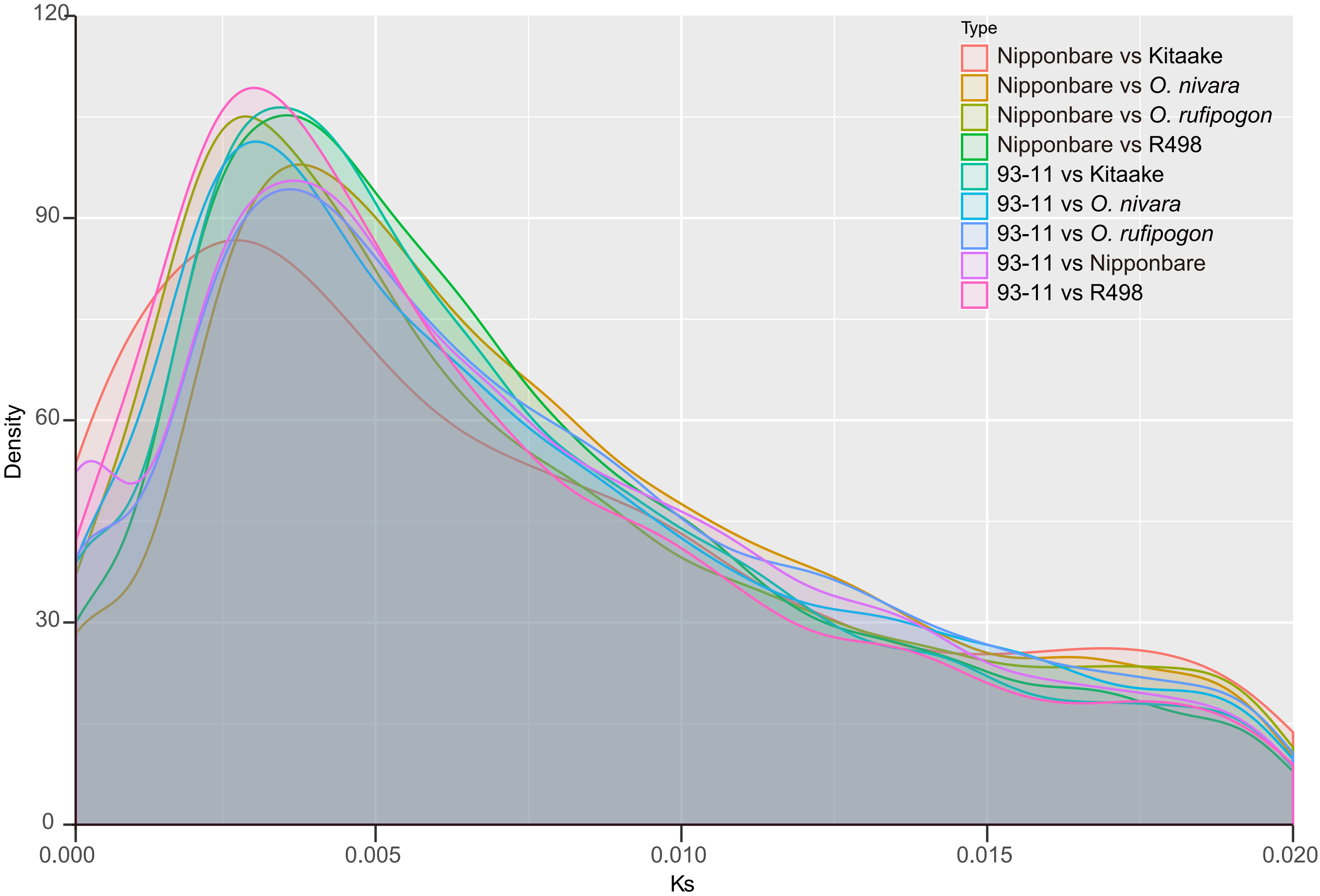

Supplementary Figure 8. Ks distribution among cultivars and wild species. The peak value of Ks distribution generated from orthologous genes between 93-11 and Nipponbare is 0.0040, which is larger than that of 93-11 vs *O. nivara* (0.0025) and Nipponbare vs *O. rufipogon* (0.0025), indicating the independent origin of the two cultivars.

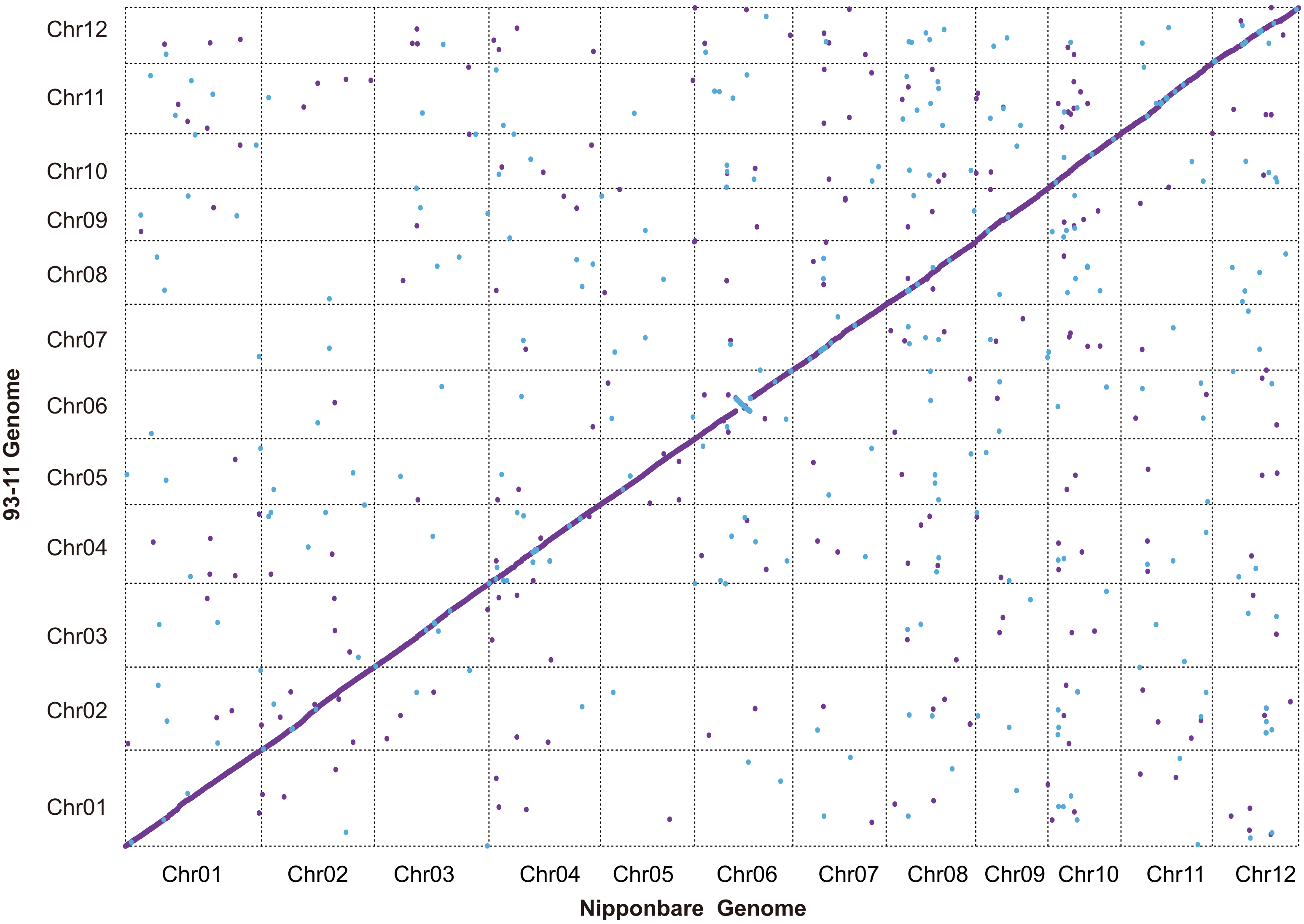

Supplementary Figure 9. Synteny comparison of 93-11 and Nipponbare. The inversion region located at the chromosome 6 was detected in the 93-11 genome.

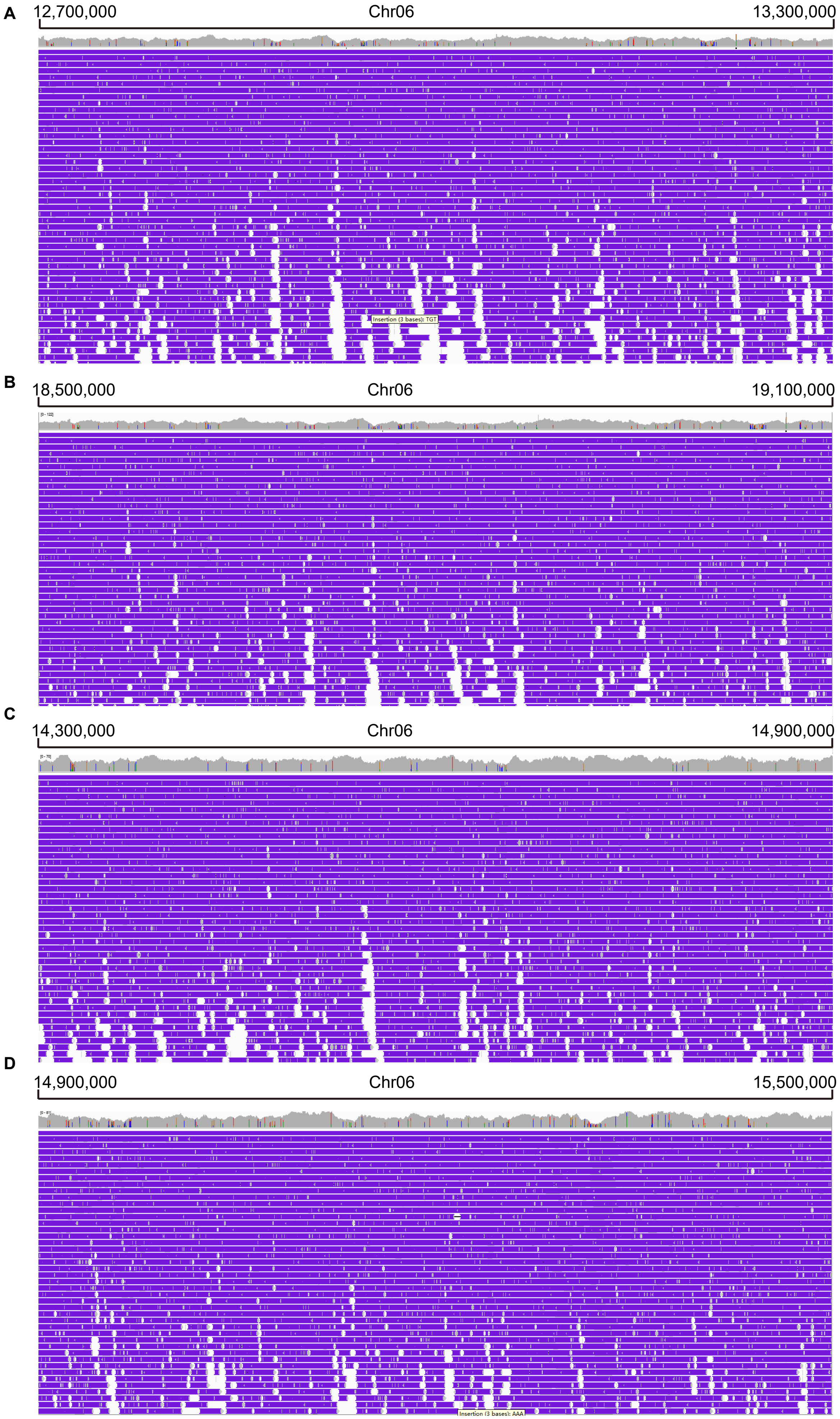

Supplementary Figure 10. Inversions in chromosome 6 confirmed by PacBio long reads. (A) and (B) are the 5' and 3' boundary of the paracentric inversion with 300kb flanking in each side, respectively. (C) and (D) are the 5' and 3' boundary of the percentirc inversion with 300kb flanking in each side , respectively. Long reads can cross the boundary, indicating the correctness of the inversion. Each continuous purple stripe represents a PacBio long read.

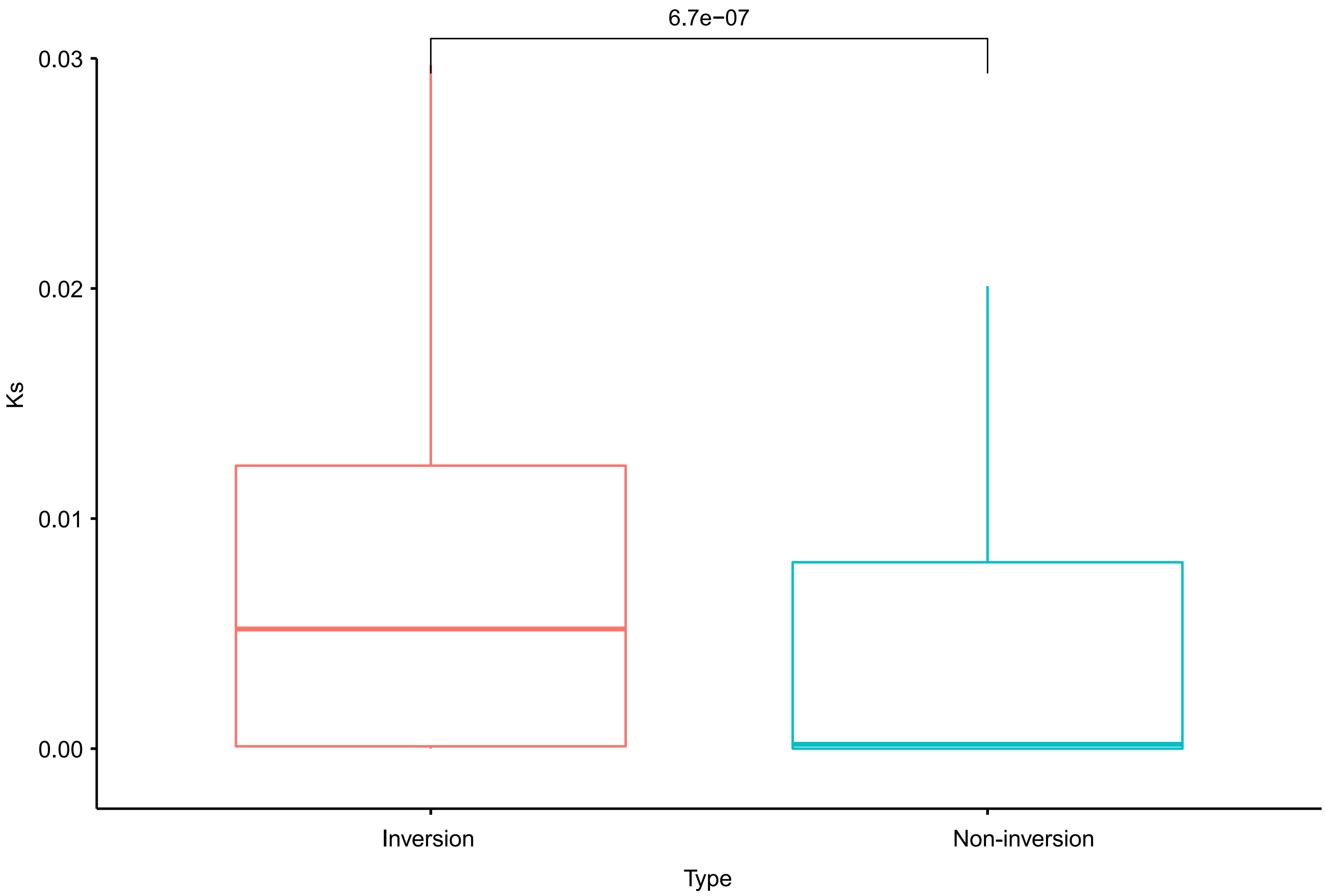

Supplementary Figure 11. Ks value comparison of homologous genes in the inversion and non-inversion region. The genes in the inversion region were evolving significantly faster than other genes (p value =6.7e-07).

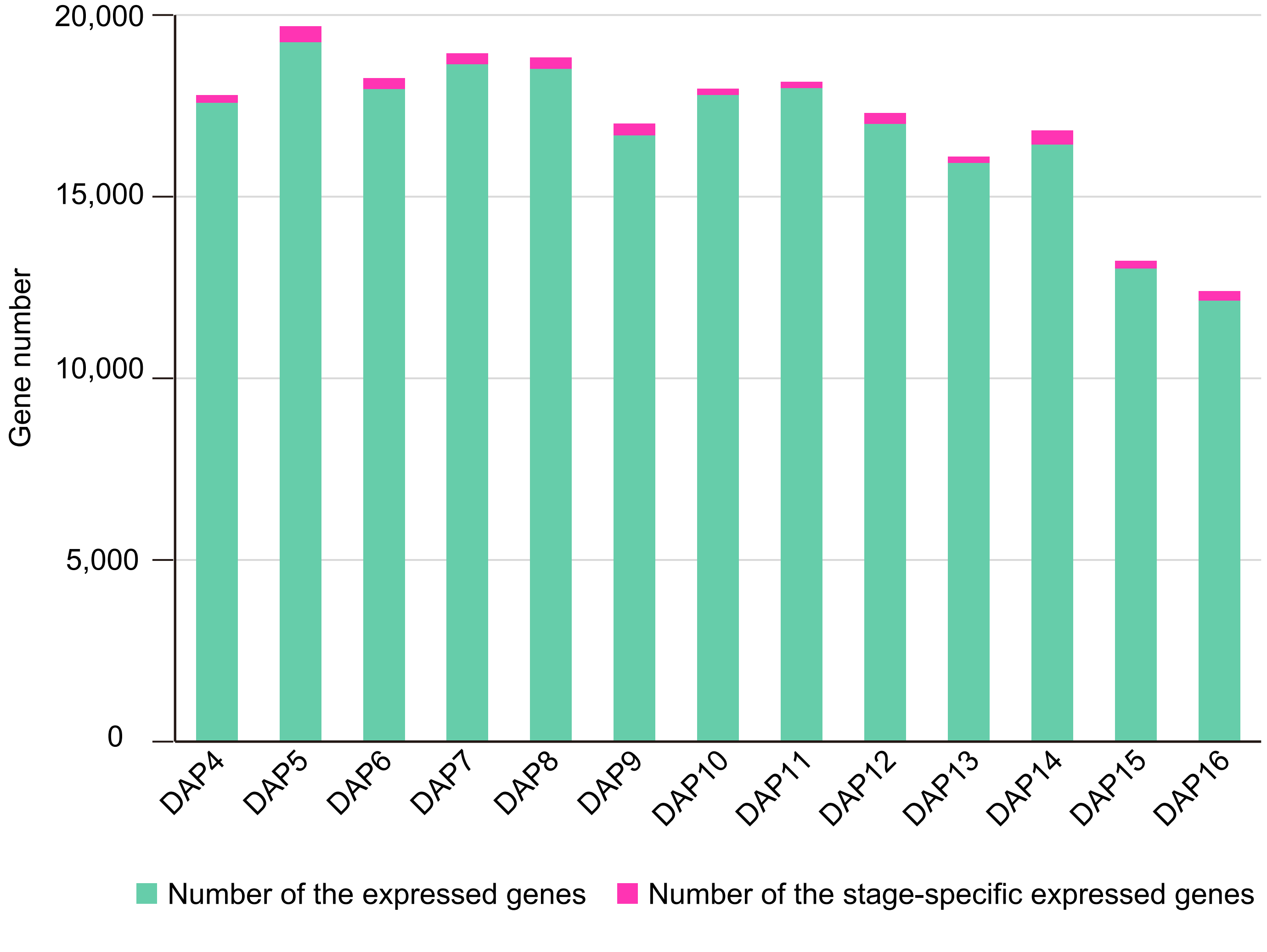

Supplementary Figure 12. Number of the expressed genes and stage-specific expressed genes at 13 stages. We generated the transcriptome data of seeds collected from 4 days after pollination (DAP) to DAP 16, and genes with TMP>=1 were considered as expressed genes. The stage-specific expressed genes were identified by Mfuzz and WGCNA.

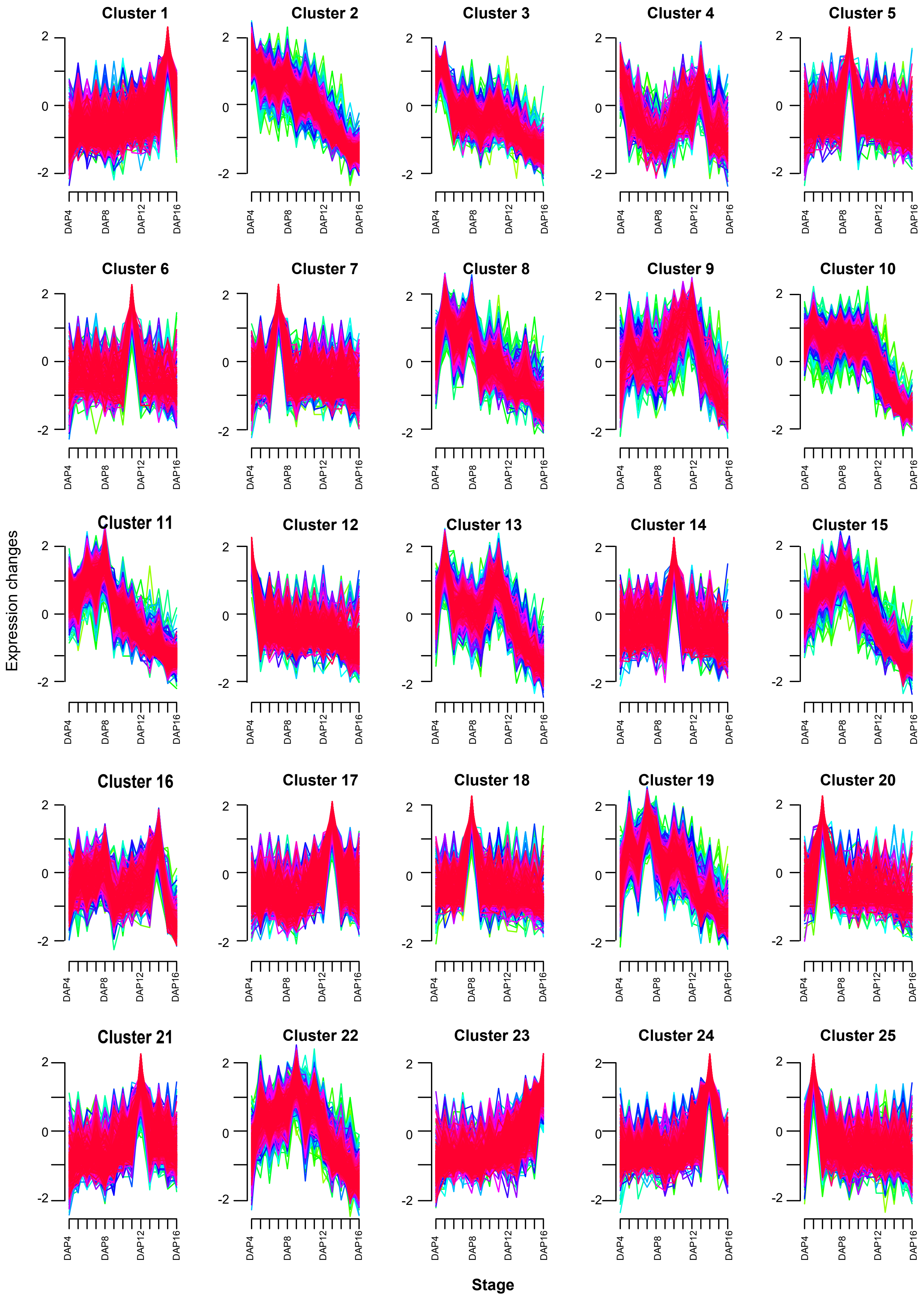

Supplementary Figure 13. Clustering analysis of gene expression at the 13 stages. A total of 25 clusters were identified by Mfuzz, and each cluster was characterized primarily by a high level of gene expression at one or more of the 13 stages.

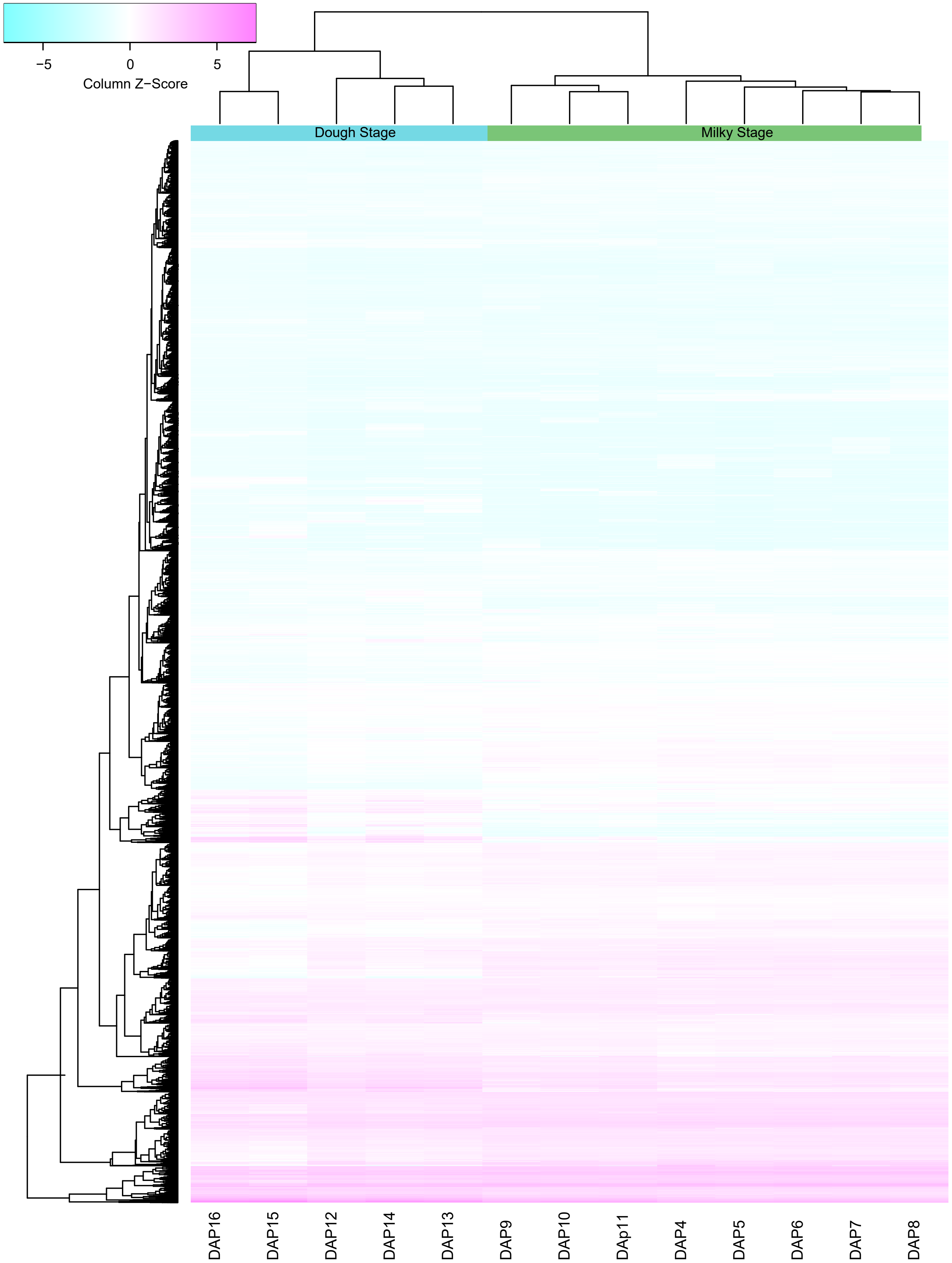

Supplementary Figure 14. Gene expression profile at the 13 stages. The expression profile of the genes expressed at least one stages. The 13 stages can be divided into two main group, corresponding to the milky and dough stages of rice seed development, respectively.

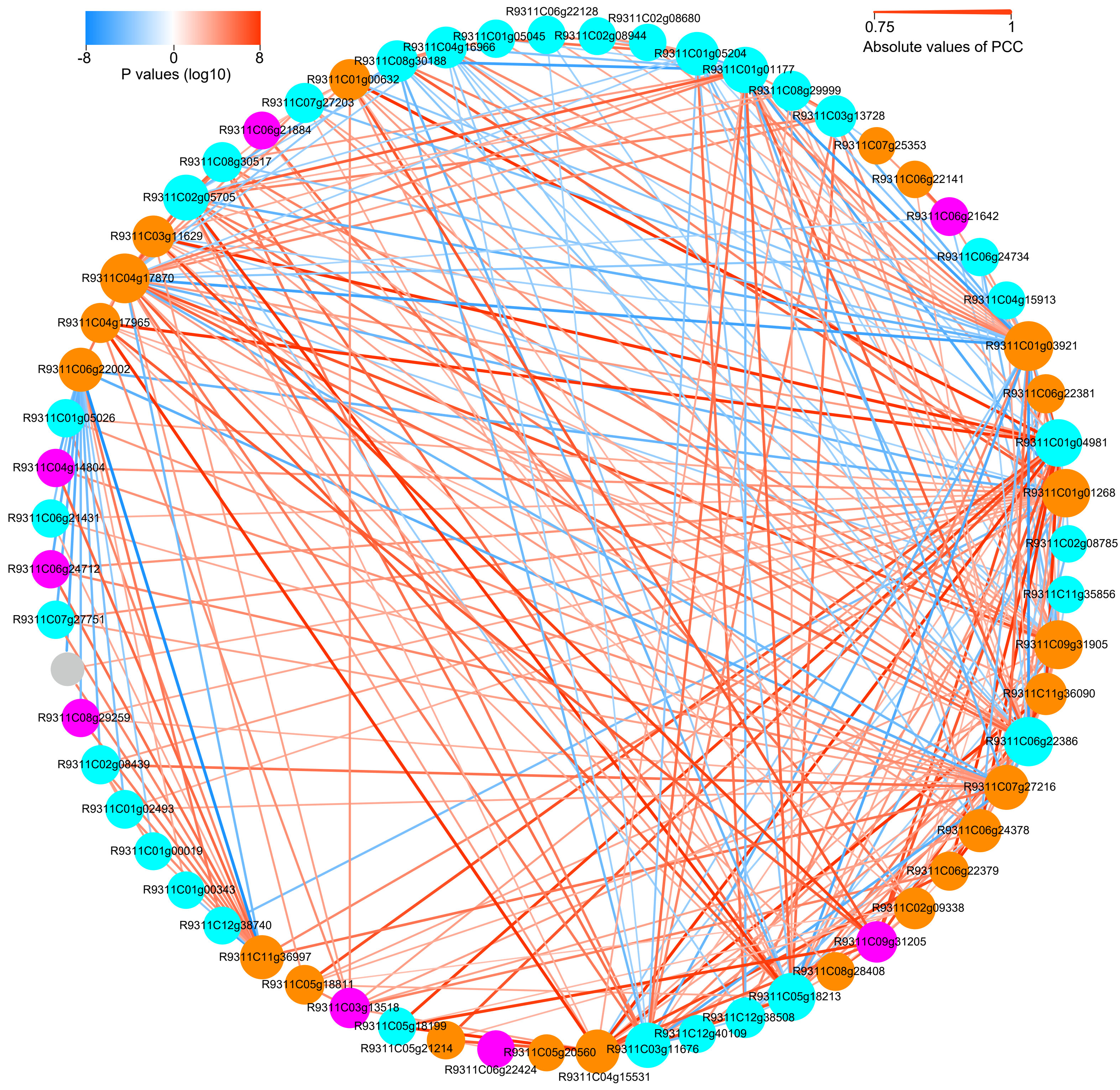

Supplementary Figure 15. Gene regulation network related to starch synthesis. The network contained 8 starch synthesis-related genes and 54 transcription factors (TF), of which 22 TFs were directly involved in regulating the expression of SSRGs. The colors pink, orange, and blue represent SSRGs, SSRG regulation-related TFs and other TFs, respectively. The color of the line represents the *p* value, and the red and blue shades represents the positive regulation and the negative regulation, respectively. The width of the line represents the correlation, the wider of the line means the higher of the correlation.

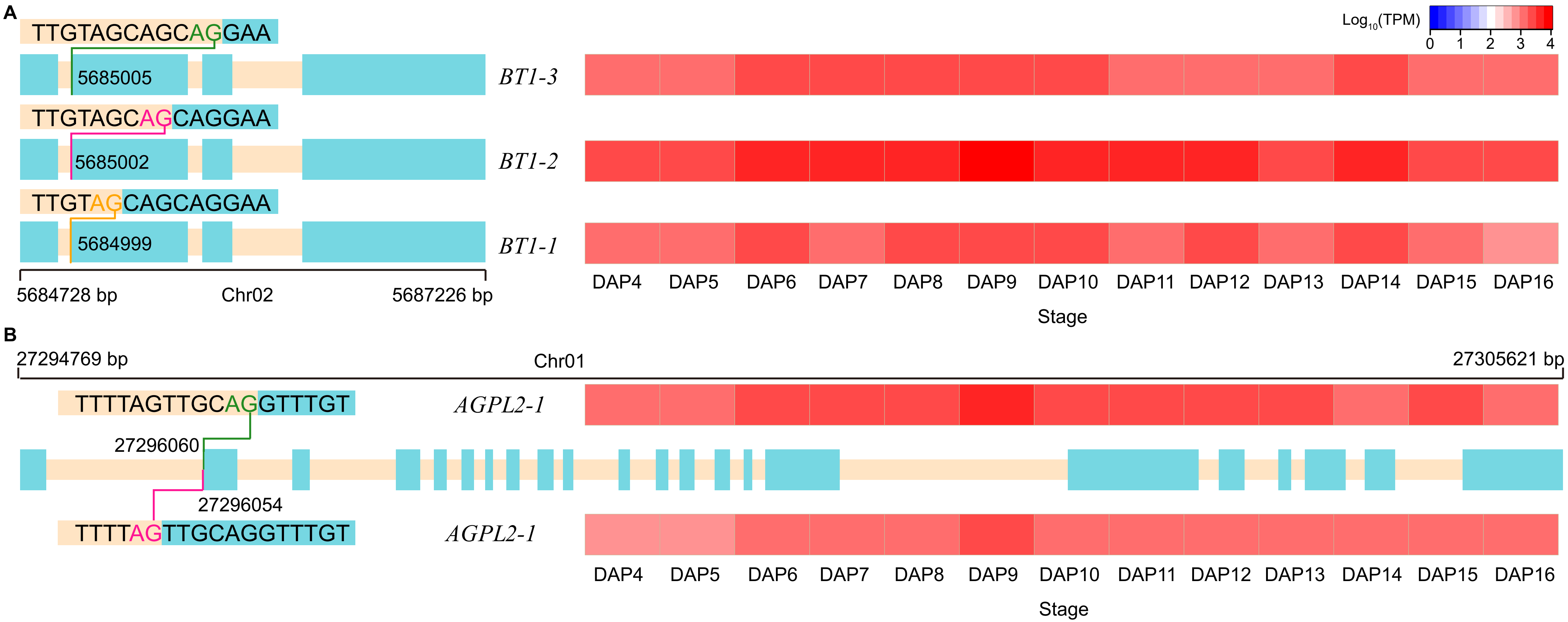

Supplementary Figure 16. Alternative splicing in the BT1 and AGPL2 genes. Three and two acceptor splice site (ASS) of the first intron in the BT1 (A) and AGPL2 (B) were detected, respectively. The splice site variation was related to multiple occurrence of the AG sequences in the 3'-ends of the first intron.
